# NbN Broadband Plasmonic Absorbers for Efficient Hot‐Carrier Injection Toward Improved Solar‐to‐Hydrogen Conversion

**DOI:** 10.1002/advs.77436

**Published:** 2026-08-24

**Authors:** Tzu‐Yu Peng, Tse‐Fu Huang, Takeshi Yamaguchi, Jia‐Wern Chen, Hsing‐Yu Kuo, Ying‐Rang Zhuang, Hung Wei Shiu, Seongmin Im, Yang Zhao, Takuo Tanaka, Ho‐Hsiu Chou, Yu‐Jung Lu

**Affiliations:** ^1^ Research Center for Applied Sciences Academia Sinica Taipei Taiwan; ^2^ Graduate Institute of Applied Physics National Taiwan University Taipei Taiwan; ^3^ Department of Chemical Engineering National Tsing Hua University Hsinchu Taiwan; ^4^ Metaphotonics Research Team RIKEN Center for Advanced Photonics Saitama Japan; ^5^ National Synchrotron Radiation Research Center (NSRRC) Hsinchu Taiwan; ^6^ Department of Electrical and Computer Engineering University of Illinois Urbana‐Champaign Urbana IL USA

**Keywords:** hydrogen evolution, localized surface plasmon resonance, metasurface, perfect absorber, transient absorption spectra, transition metal nitride

## Abstract

Solar‐driven hydrogen evolution offers a promising route to sustainable fuel production, yet conventional thin‐film photocatalysts suffer from narrow absorption and inefficient carrier utilization. Here, we demonstrate that niobium nitride (NbN) broadband plasmonic metasurface absorbers (MA) serve as an efficient hot‐carrier injection platform for solar energy conversion. Specifically, the quasi‐epitaxial NbN has a low work function (4.0 eV), which reduces the interfacial injection barrier and facilitates hot‐carrier transfer into the photocatalyst. Meanwhile, the NbN MA exhibits broadband visible absorption (>80%), and its resonances generate local hot spots that enhance interfacial light–matter interactions, boosting hot‐carrier generation and injection. Transient absorption spectroscopy further reveals sub‐picosecond hot‐carrier generation (250 fs). As a result, when coupled with the polymer photocatalyst PFBPO, the NbN metasurface achieves a remarkable 481% increase in the hydrogen evolution rate at 500 nm, accompanied by an ultrashort interface hot‐carrier transfer lifetime of 1.9 ps, extending the photoresponse far beyond the intrinsic absorption of the polymer. Notably, we observed a record‐high apparent quantum yield of 4.1% at 460 nm. These findings highlight the critical role of non‐thermal hot‐carrier injection in plasmonic catalysis in driving interfacial charge transfer and highlight NbN‐based metasurfaces as a robust platform for efficient solar‐to‐chemical energy conversion.

## Graphical abstract

Niobium nitride (NbN) broadband plasmonic metasurface absorbers provide an efficient platform for solar‐energy conversion through hot‐carrier injection. Quasi‐epitaxial NbN features a low work function of 4.0 eV, reducing the interfacial injection barrier and facilitating charge transfer into adjacent photocatalysts. Combined with broadband visible absorption and localized plasmonic hot spots, this platform promotes efficient solar‐to‐chemical conversion for sustainable fuel production applications.

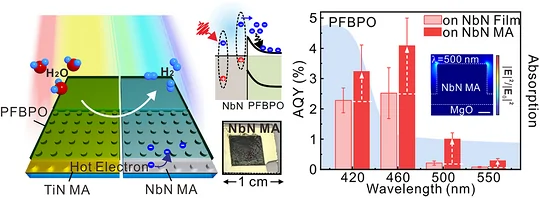

## 1 Introduction

Solar‐driven hydrogen evolution through photoelectrochemical (PEC) or photocatalytic water splitting has long been considered a promising pathway toward sustainable solar fuel production [1, 2, 3, 4, 5, 6, 7]. As the most abundant renewable energy source on Earth, solar energy has received increasing attention for a broad range of applications [5], including water splitting for hydrogen evolution [4, 7, 8, 9, 10], hydrogen dissociation [11, 12, 13], CO_2_ reduction [14, 15], desalination [16], and nitrogen fixation for ammonia synthesis [17]. In recent decades, diverse semiconductor systems, including metal oxides, nitrides, sulfides, and polymeric photocatalysts, have been explored as photocatalytic films [18, 19, 20]. However, most thin‐film photocatalysts suffer from limited optical absorption within the solar spectrum because of their intrinsically narrow absorption bands or wide bandgaps, leading to inefficient solar‐to‐fuel conversion (with a solar‐to‐hydrogen (STH) conversion efficiency exceeding 1%) [6, 18, 19, 20]. Strategies to enhance solar energy harvesting, such as bandgap engineering and heterojunction construction, have achieved progress but remain constrained by stability, scalability, and carrier recombination losses. In particular, current photocatalytic hydrogen evolution still faces the following persistent bottlenecks: the mismatch between narrow semiconductor bandgaps and the broad solar spectrum, rapid charge recombination at interfaces, and degradation of catalytic films under prolonged operation in aqueous environments. These limitations highlight the urgent need for new material platforms that simultaneously combine broadband optical absorption, efficient carrier dynamics, and long‐term durability.

Plasmonics and metasurfaces have emerged as a complementary pathway to overcome these challenges [21, 22, 23, 24, 25, 26, 27]. Metallic nanostructures support localized surface plasmon resonances (LSPRs) [28, 29, 30], which concentrate light into subwavelength volumes and enable both enhanced broadband absorption and the generation of energetic nonequilibrium carriers [24, 31, 32]. Importantly, the nonradiative decay of plasmons produces hot carriers on ultrashort timescales (<50 fs) via Landau damping and electron–electron scattering [24, 31, 32], with lifetimes of several hundred femtoseconds before thermalization. Their energy distribution deviates from Fermi–Dirac statistics and may even exceed the photon energy due to impact excitation and Auger heating [24, 32], offering unique driving forces for interfacial charge transfer. Such carriers can overcome kinetic barriers and enable photochemical reactions otherwise inaccessible within the band edge limits of semiconductors. Recent studies have highlighted both the opportunities and the paradoxes of hot‐carrier versus thermal effects in plasmon‐assisted photocatalysis [23, 24, 25, 33, 34], and have demonstrated all‐plasmonic water splitting and antenna–reactor designs for selective solar‐to‐fuel conversion [21, 22, 35]. For instance, junction‐based measurements have enabled direct probing of hot‐carrier transport across plasmonic interfaces [33, 34].

While noble metals (Au and Ag) are common plasmonic materials for hot‐electron [34] or hot‐hole injection [33] and photocatalytic enhancement [36, 37, 38], their poor thermal stability and the intrinsically high quality factor of their LSPRs limit their effectiveness as broadband absorbers [39]. Refractory plasmonic materials, such as transition‐metal nitrides (TiN, HfN, and NbN), have thus attracted attention because of their combination of metallic optical response, chemical stability, and high melting points [40, 41, 42, 43, 44, 45]. Unlike noble metals, these ceramic plasmonic materials exhibit superior resistance against oxidation and morphological degradation, maintain their plasmonic response under harsh thermal and chemical environments, and offer wideband optical absorption that better matches the solar spectrum [40, 43, 44, 45, 46]. Owing to the relatively low quality factor of LSPRs in TMNs, broader plasmonic resonances are enabled, which are advantageous for broadband visible light harvesting [39, 46]. From an atomic perspective, the incorporation of nitrogen atoms into the parent metal structure induces lattice expansion due to the formation of metal–nitrogen bonds. This structural change leads to a contraction of the metal d‐band, resulting in an increased density of states (DOS) near the Fermi level [47]. The increased DOS provides more occupiable states for photogenerated carriers, thereby facilitating their efficient electron‒hole separation and carrier transport, which is critical for improving catalytic production [35]. These features make NbN an appealing candidate for plasmonic and photocatalytic applications beyond cryogenic superconducting devices [48].

Here, we demonstrate that a NbN metasurface absorber (MA) functions as a highly efficient plasmonic hot‐carrier platform for solar‐driven hydrogen evolution. By using quasiepitaxial NbN films to fabricate a large‐area metasurface structure, we realize broadband visible absorption (>80%) spanning 400–750 nm, together with strong field confinement that greatly enhances photoexcited carrier generation and hot‐carrier injection. Ultrafast pump–probe transient absorption spectroscopy (TAS) reveals subpicosecond relaxation dynamics (250 fs) and accelerated interfacial carrier transfer compared with those of unpatterned NbN, thus confirming the role of plasmon‐enhanced hot carriers. When integrated with the polymer photocatalyst PFBPO, the NbN absorber yields a 481% increase in the hydrogen evolution rate at 500 nm which is well beyond the intrinsic absorption window of the polymer. The measured apparent quantum yield (AQY) of ∼ 4.1% at 460 nm can be observed. These findings highlight that plasmon‐derived hot carriers (non‐thermal effect), rather than mere photothermal heating [27, 49], actively drive the hydrogen evolution reaction. This study not only demonstrates the plasmonic functionality of NbN metasurfaces but also provides mechanistic insight into the correlation between hot‐carrier generation, interfacial charge injection, and solar hydrogen evolution in a refractory plasmonic metasurface system. Building on previous important studies of TiN‐based refractory plasmonic photocatalysts [35], our work extends this concept to broadband NbN MA and combines ultrafast spectroscopy with photocatalytic measurements to clarify the role of plasmon‐generated hot carriers in enhancing hydrogen evolution. By bridging ultrafast spectroscopy with catalytic measurements, our work fills the critical knowledge gap in understanding hot‐carrier dynamics in transition‐metal nitrides, paving the way for durable, broadband, and scalable plasmonic platforms for solar‐to‐fuel conversion. Beyond hydrogen generation, the demonstrated paradigm may be extended to CO_2_ reduction, ammonia production, and selective organic transformations, bridging nanophotonic light management with interfacial hot‐carrier photocatalysis to accelerate next‐generation energy conversion technologies [15, 50, 51, 52].

## 2 Results and Discussion

A schematic of the broadband MA designed for solar energy harvesting is shown in Figure 1a. (Detailed information on the fabrication process of the nitride‐based MA is provided in the Methods section and shown in Figure S1). To realize a large‐area, lithography‐compatible platform, we deposited high‐quality TiN and NbN thin films by reactive magnetron sputtering at 800°C (see Table S1 and Table S2). Figure 1b and Figure S2 show the measured dielectric permittivity of the optimized TiN and NbN films. On the basis of our previous findings regarding the strong substrate selectivity of transition‐metal nitrides, MgO(100) was chosen to promote quasi‐epitaxial growth under these high‐temperature conditions [48]. This approach yields uniform films with excellent crystalline quality and atomically smooth surface roughness (RMS∼0.1 nm), as confirmed by XRD and AFM measurements (Figure 1c and Figure S3). The XRD pattern shows a sharp (200) diffraction peak at 40.8°, confirming the quasi‐epitaxial growth of the NbN film with a preferred (100) orientation. The inset shows the measured work function of the NbN (100) film from UPS, which is 4.0 eV. The dielectric permittivity results show that both the 100 nm‐thick NbN and TiN films exhibit optically metallic behavior (Re(ε) < 0) across the visible–near‐IR region. Notably, NbN displays a pronounced blueshift of the epsilon‐near‐zero (ENZ) point to 320 nm, compared with 490 nm for TiN, indicating that it has a higher bulk plasma frequency and free‐carrier concentration. As a result, NbN combines a more negative real permittivity with lower imaginary loss, enabling it to sustain strong LSPRs at shorter wavelengths. These characteristics render NbN particularly advantageous for broadband light harvesting and hot‐carrier generation, both of which are critical for efficient solar‐to‐fuel conversion. Finite‐difference time‐domain (FDTD) simulations and experimental measurements confirm that a periodic NbN nanodisk metasurface achieves an average absorption >80% across the visible spectrum (Figure 1d). Increasing the nanodisk diameter induces a systematic redshift of the absorption peak toward longer wavelengths, which is consistent with the LSPR mode of the designed nanostructures. The metasurface design also incorporates a thin reflective back reflector to suppress transmission and maximize light–matter interaction within the designed plasmonic platform. OM and SEM images of the NbN MA with disk diameters ranging from 190 to 220 nm (bottom to top: 190, 200, 210, and 220 nm; height of 110 nm and period P of 330 nm) are shown in Figure 1e and Figure S4, respectively. The simulated electric‐field distributions (Figure 1f) reveal highly localized corner hot spots. At 400 nm, NbN exhibits stronger localized electromagnetic fields than TiN, which can be attributed to its higher bulk plasma frequency. Notably, although NbN exhibits a larger imaginary permittivity than TiN at approximately 650 nm, the optimized NbN nanodisks still generate stronger localized fields than the TiN nanodisks at this wavelength. This result indicates that the local‐field enhancement is governed not only by the intrinsic dielectric properties of the material but also by the nanodisk geometry, substrate configuration, and light–nanostructure coupling. Consequently, the optimized NbN MA supports broadband plasmonic absorption and localized electromagnetic hot spots across the visible spectrum, facilitating hot‐carrier generation and interfacial injection into the adjacent semiconductor photocatalyst.

**FIGURE 1:**
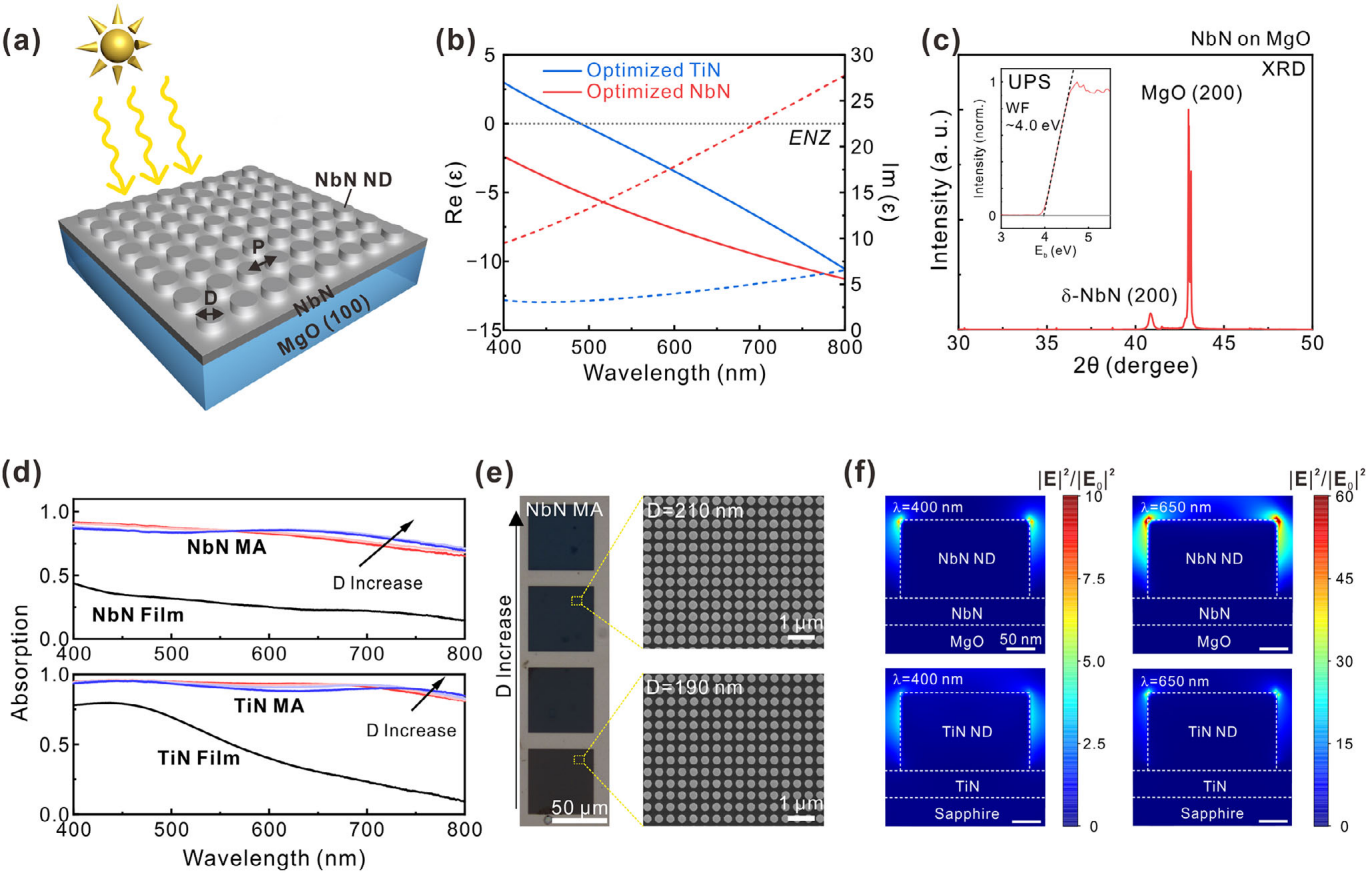
Broadband NbN plasmonic metasurface absorber (MA). (a) Schematic of the NbN MA on an MgO(100) substrate, comprising a periodic NbN nanodisk (ND) array atop a 50‐nm‐thick NbN back‐reflector, forming an LSPR for broadband absorption. (b) Measured dielectric permittivity of optimized TiN and NbN thin films, showing metallic behavior (Re (ε) < 0) across the visible spectrum and a blueshifted ENZ point for NbN (320 nm) compared to TiN (490 nm). (c) XRD pattern confirming quasiepitaxial growth on MgO(100). Inset: UPS spectrum showing a measured work function of 4.0 eV. (d) Simulated absorption spectra of NbN and TiN metasurfaces with varying nanodisk diameters, compared with unpatterned films. Both NbN and TiN MA exhibit broadband absorption exceeding 80% across the visible region. (e) OM and SEM images of fabricated NbN metasurfaces with disk diameters of 210 and 190 nm, demonstrating large‐area uniformity and precise controllable nanopatterning. (f) Simulated electric‐field distributions at λ = 400 and 650 nm for NbN and TiN MAs, showing stronger hot‐spot localization in NbN due to its higher bulk plasma frequency.

To understand the photoexcited carrier dynamics in the NbN and TiN systems, we performed femtosecond pump–probe TAS [53, 54]. Figure 2a displays the transient absorption maps as a function of time and wavelength for the NbN and TiN thin films as well as their MAs. The transient absorption spectra of these samples from 0.1 to 10 ps are shown in Figure S5. Among all the samples, the NbN MA exhibits the most intense and spectrally broad transient bleaching, signifying pronounced suppression of ground‐state absorption and efficient hot‐carrier generation. Such bleaching is due to the LSPR‐induced redistribution of electronic populations, which transiently affects the complex permittivity. The transient spectra extracted at a delay of 0.5 ps (Figure 2b) reveal that compared with the unpatterned NbN film, the NbN MA not only enhances the overall bleaching amplitude but also broadens the spectral response. This spectral broadening indicates that the hot‐carrier distribution remains highly nonthermal at early times, extending across a wide energy window. This observation is consistent with plasmon‐assisted generation of energetic electrons and holes beyond the thermalized Fermi–Dirac distribution [24].

**FIGURE 2:**
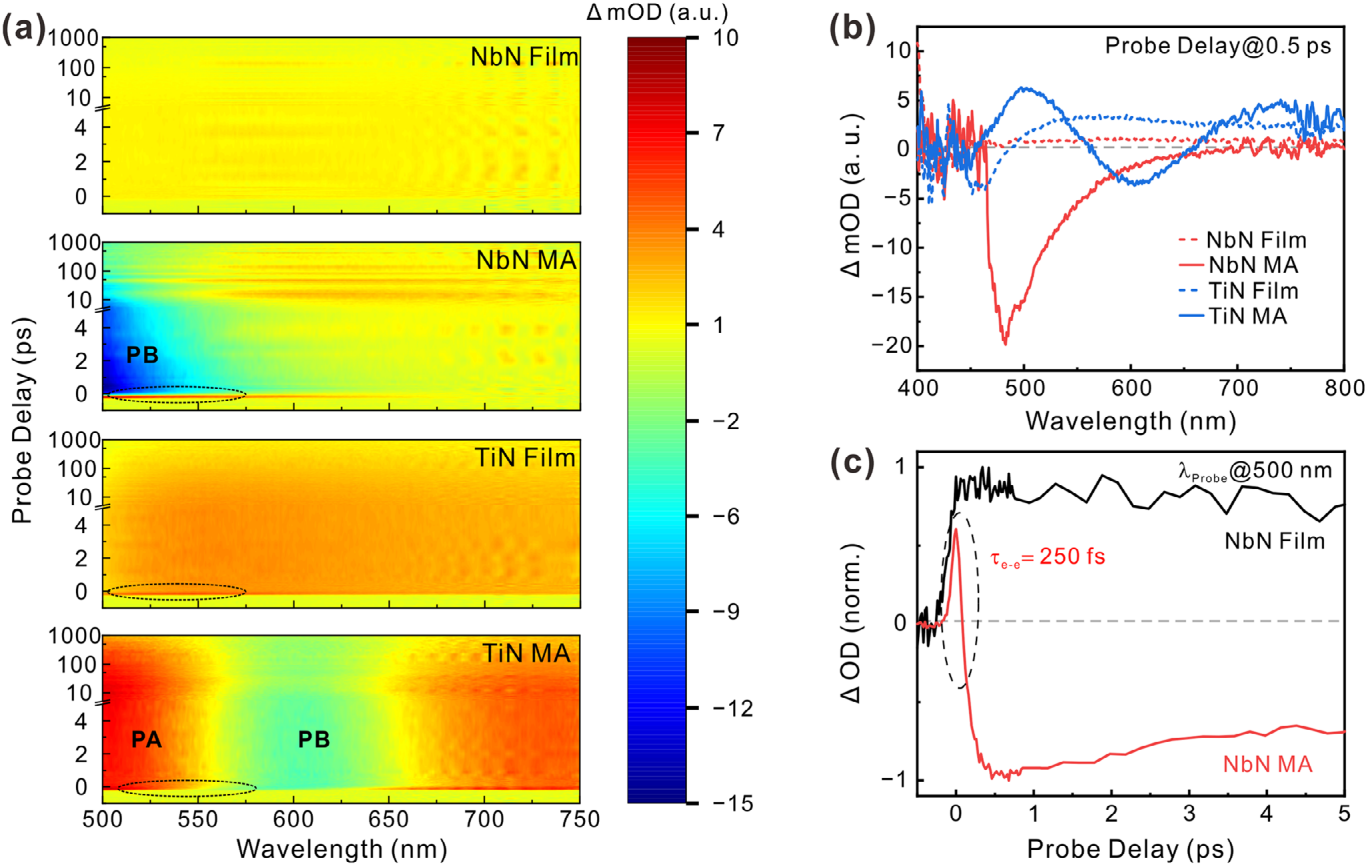
Ultrafast carrier dynamics in NbN and TiN films and metasurface absorbers. (a) Pump–probe transient absorption (TAS) maps as a function of time and wavelength for pristine NbN and TiN films and their metasurface absorbers (MAs). The NbN MA exhibits the strongest and broadest transient bleaching, indicating efficient photoexcited carrier generation and energy redistribution. (b) Transient spectra at a probe delay of 0.5 ps, where the NbN MA exhibits both stronger bleaching amplitude and broader spectral response compared with the unpatterned NbN film, indicative of a nonthermal hot‐carrier distribution. TiN samples display weaker and narrower responses. (c) Normalized kinetic traces probed at 500 nm, revealing a significantly faster carrier relaxation lifetime (250 fs) in the NbN MA relative to the pristine film, consistent with enhanced electron–electron scattering in localized hot spots.

In contrast, TiN‐based samples exhibit less bleaching with a smaller bandwidth at 600 nm, suggesting a reduced hot‐carrier density and faster thermalization dynamics. The kinetic traces (Figure 2c) at a probe wavelength of 500 nm further reveal a sub‐picosecond lifetime (250 fs) for the NbN MA, which is notably shorter than that of the pristine NbN film. At this stage, the dynamics originate solely from the intrinsic plasmonic resonance of NbN, without any contribution from semiconductor charge transfer. The accelerated relaxation can thus be attributed primarily to enhanced electron–electron scattering within localized plasmonic hot spots, where elevated carrier densities facilitate rapid energy redistribution. Accordingly, the observed ultrafast decay is governed by intrinsic carrier cooling in NbN, which is distinct from later‐stage processes involving interfacial hot‐carrier transfer. Taken together, these results establish the NbN MA as a promising platform for driving ultrafast PEC reactions.

To directly probe interfacial charge transfer between the NbN MA and the polymer photocatalyst PFBPO (Figure S6) [55], we performed femtosecond pump–probe TAS measurements again. Figure 3a shows photographs of PFBPO polymer films integrated with NbN and TiN films and their MAs, revealing uniform polymer coverage over a subcentimeter‐scale area (0.6 × 0.6 cm^2^) under UV illumination. This ensures consistent interface quality across the optically pumped region. The TAS dynamics of PFBPO integrated with the planar NbN film and NbN MA are shown in Figure 3b,c and Figure S7, respectively. Upon photoexcitation, compared with its planar counterpart, PFBPO on the NbN MA exhibits a significantly enhanced transient signal and accelerated rise and decay dynamics. The energy band diagrams (Figure 3d) clarify the plasmon‐enhanced hot‐carrier transfer at the interface of the transition metal nitride plasmonic structure and PFBPO. Compared with PFBPO's highest occupied (HOMO) and lowest unoccupied molecular orbital (LUMO) levels, which define a bandgap of 2.69 eV, NbN exhibits a lower work function (4.0 eV) relative to TiN (4.9 eV) as measured by UPS. Upon reaching thermal equilibrium at the heterojunction, the band alignment induces band bending, and interface charge trapping generates a built‐in electric field at the interface. The interfacial energy‐level alignment plays a critical role in hot‐carrier injection into the polymer. Owing to its lower work function, NbN forms a more favorable energy‐level alignment with PFBPO than TiN, reducing the interfacial barrier and enabling more efficient hot‐electron injection into the polymer layer. To experimentally examine the interfacial potential differences, we performed Kelvin probe force microscopy (KPFM) measurements on PFBPO‐coated NbN and TiN substrates (Figure S8). A selected region of each PFBPO film was removed to expose the underlying substrate, allowing direct comparison of the surface‐potential contrasts between the PFBPO‐covered and bare substrate regions. The KPFM potential maps and corresponding line profiles reveal a smaller surface‐potential contrast for NbN (approximately 56.2 mV) than for TiN (approximately 90.5 mV). This smaller contrast is consistent with a reduced energy‐level mismatch for hot‐electron injection at the NbN/PFBPO interface. This nonthermal distribution of carriers, generated during the <100 fs plasmon decay, maximizes the interfacial charge transfer efficiency before electron–electron scattering thermalizes the carrier ensemble. The strong bleaching and rapid growth kinetics reflect efficient LSPR‐enhanced hot‐carrier injection, which is further corroborated by the presence of a broad positive photoinduced absorption band in the spectra at a probe delay of 1 ps (Figure 3e). This positive signal is attributed to the population of PFBPO excited states via hot‐carrier transfer from the NbN MA, which is consistent with a charge‐transfer‐induced excited‐state absorption mechanism. In comparison, the TiN MA exhibits a relatively broad photobleaching signal around 640 nm. This feature is primarily associated with the plasmonic response of the TiN nanostructures, such as LSPR‐induced carrier redistribution. The markedly different behavior between the two metasurfaces in Figure 3e can be attributed to the lower work function of NbN, which produces a more favorable NbN/PFBPO energy alignment, which reduces the interfacial barrier for hot‐electron injection into PFBPO. In contrast, the higher work function of TiN leads to a larger potential barrier, limiting the efficiency of hot‐carrier transfer despite the presence of plasmonic nanostructures. Fitted time‐resolved kinetics (Figure 3f and Table S3) at a probe wavelength of 730 nm using a two‐exponential decay model reveal that the hot‐carrier injection time constant at the interface of the PFBPO/NbN MA heterostructure is 1.9 ps, nearly 35 times faster than that of the PFBPO/Si interface (69 ps). This dramatic acceleration can be attributed to two effects: (1) the near‐field hot spot enhancement, which locally increases the excitation rate and photoexcited carrier density, and (2) the more favorable interfacial band alignment between NbN/PFBPO, which reduces the effective barrier height (Schottky barrier lowering). The TiN control samples exhibit weaker signals and slower injection kinetics, consistent with their lower hot‐carrier generation efficiency. These results conclusively demonstrate that the NbN MA not only increases hot‐carrier generation but also facilitates rapid interfacial charge separation, laying the foundation for high‐efficiency solar‐to‐chemical energy conversion in solar‐driven hydrogen evolution systems.

**FIGURE 3:**
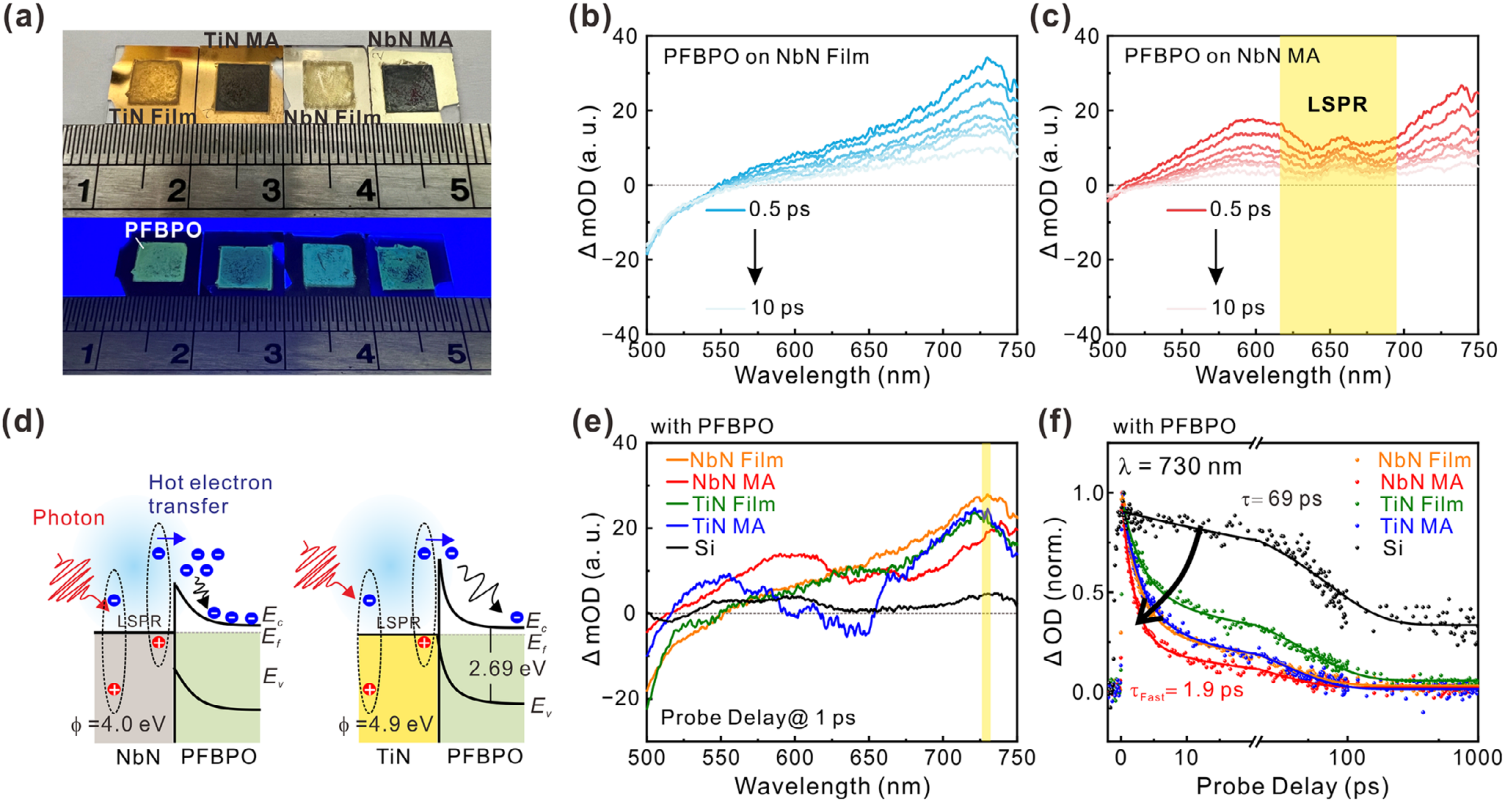
Ultrafast plasmonic hot‐carrier injection in the PFBPO/NbN metasurface absorber (MA). (a) Photographs of the PFBPO‐coated TiN film, NbN film, and their MAs; lower panel shows uniform coating regions (0.6 × 0.6 cm^2^) under UV illumination. (b,c) Transient absorption spectra of PFBPO on the NbN film and NbN MA, revealing markedly enhanced and faster transient response in the presence of LSPR. (d) Schematic band alignment for NbN/PFBPO and TiN/PFBPO heterostructures, highlighting LSPR‐driven efficient hot‐carrier transfer pathway in the NbN MA. (e) Comparison of transient spectra at a 1 ps probe delay for PFBPO on NbN and TiN films and their MAs, benchmarked against PFBPO on a Si reference, showing the strongest photoinduced positive absorption in the PFBPO/NbN MA. (f) Kinetic traces probed at 730 nm showing accelerated carrier relaxation in the PFBPO/NbN MA (τ ∼1.9 ps) compared with the PFBPO/NbN film (τ ∼ 69 ps) and other control samples, evidencing efficient plasmonic hot‐carrier extraction and ultrafast interfacial transfer due to the interface electric field.

To quantitatively assess the impact of plasmonic hot‐carrier injection on solar‐driven hydrogen production, we performed photocatalytic hydrogen evolution measurements under AM 1.5G simulated sunlight irradiation (Figure 4a and Figure S9). The incident light intensity was calibrated to 1 Sun (1000 W m^−2^), and all measurements were conducted under ambient conditions. Triethylamine (TEA) was selected as the sacrificial hole scavenger after systematic optimization, while MeOH was added to prevent phase separation between water and TEA. Hydrogen gas evolution was monitored every 30 min via gas chromatography (GC) with a thermal conductivity detector (TCD). The hydrogen evolution rates (HERs) for PFBPO deposited on Si, TiN film, TiN MA, NbN film, and NbN MA are summarized in Figure 4b. The corresponding HERs were 2.11, 2.67, 4.06, 3.37, and 7.34 mmol  g^−^^1^ h^−^^1^, respectively. Remarkably, the NbN MA achieves an HER of 7.34 mmol g^−^^1^ h^−^^1^, corresponding to a 218% increase over the unpatterned NbN film and a 349% increase relative to the Si reference. Similarly, compared with the Si substrate, the TiN MA shows a 193% enhancement, validating the generality of the plasmonic resonance approach. The superior performance of plasmonic NbN relative to that of plasmonic TiN can be directly correlated with its higher plasma frequency and more negative real part of the permittivity, which support stronger hot spots and spectrally broader localized surface plasmon resonances (LSPRs), thereby increasing the plasmonic hot‐carrier generation yield. Notably, the MA geometry introduces intense near‐field hot spots, which not only amplify the excitation rate but also accelerate interfacial hot‐carrier injection into the PFBPO layer, as revealed by TAS measurements (see Figures 2 and 3). Besides, the calculated surface temperature distribution of the NbN MA under AM 1.5 illumination shows negligible temperature variation (Figure S10). To further evaluate the possible contribution of photothermal heating, we performed power‐dependent TAS measurements of PFBPO on the NbN MA. The normalized transient absorption decay profiles exhibit nearly identical carrier‐relaxation dynamics over the measured excitation‐power range (Figure S11 and Table S4). The absence of a measurable power‐dependent change in the carrier lifetimes indicates that photothermal heating is not the dominant mechanism. Together, the calculated temperature distribution and power‐dependent transient absorption measurements support the conclusion that the enhanced photocatalytic performance arises primarily from plasmon‐generated hot‐carrier injection. As a result, the combination of broadband absorption, strong local field enhancement, and ultrafast hot‐carrier injection maximizes the fraction of photoexcited carriers that successfully participate in the hydrogen evolution reaction rather than undergoing thermalization or other recombination losses. To verify this concept, the time‐dependent hydrogen generation profiles (Figure 4c) confirm the stability of the reaction rate enhancement over the entire reaction period, indicating that the NbN MA remains structurally and chemically robust under continuous irradiation and in contact with the aqueous reaction medium. These results highlight the potential of the plasmonic NbN MA as a stable and scalable platform for efficient and durable solar‐to‐hydrogen energy conversion.

**FIGURE 4:**
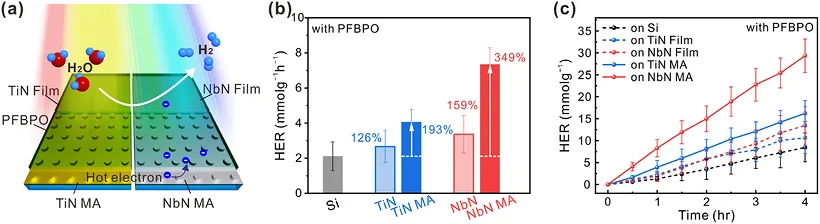
Plasmon‐enhanced solar‐driven hydrogen evolution enabled by the NbN MA. (a) Schematic illustration of solar‐driven hydrogen evolution in PFBPO films integrated with conductive nitride thin films and their MAs, where the metasurface enhances light harvesting and accelerates carrier injection. (b) Hydrogen evolution rates (HERs) of PFBPO on the TiN film, TiN MA, NbN film, and NbN MA. The TiN MA shows a 193% enhancement, while the NbN MA yields a 349% increase relative to PFBPO/silicon, demonstrating superior plasmonic hot‐carrier injection efficiency. (c) Time‐dependent HER curves on different substrates, with the NbN MA consistently showing the highest activity, confirming the synergistic effect of broadband plasmonic absorption and ultrafast hot‐carrier injection in enhancing photocatalytic performance.

To clarify the role of the NbN MA in enhancing hydrogen evolution and to evaluate the influence of spectral matching, we compared two polymer photocatalysts, PFBPO and PBDTTSO [56], that are coated on the NbN MA under identical experimental conditions. As shown in Figure 5a, PBDTTSO has a broader intrinsic absorption profile that extends well into the visible region, whereas PFBPO has a relatively narrow absorption band and weaker visible‐light absorption. This difference allows us to evaluate whether the NbN MA can enhance photocatalytic activity in spectral regions where the polymer itself absorbs less efficiently. As shown in Figure 5b, both polymers exhibit higher hydrogen evolution rates on the NbN MA than on the corresponding Si reference substrates, indicating that the enhancement is not limited to a single polymer system. Notably, the relative enhancement is substantially greater for PFBPO than for PBDTTSO. This result suggests that the NbN MA does not simply amplify the intrinsic absorption of the polymer. Instead, its broadband plasmonic absorption enables visible‐light harvesting and hot‐carrier generation, followed by interfacial carrier injection into the polymer to promote hydrogen evolution. The comparison between PFBPO and PBDTTSO therefore supports the proposed plasmon‐induced hot‐carrier mechanism and highlights the potential of NbN metasurfaces as a versatile platform for enhancing solar‐driven photocatalysis. Although the present study focuses on polymer photocatalysts, this strategy may also be extended to other semiconductor materials with suitable interfacial energy‐level alignment.

**FIGURE 5:**
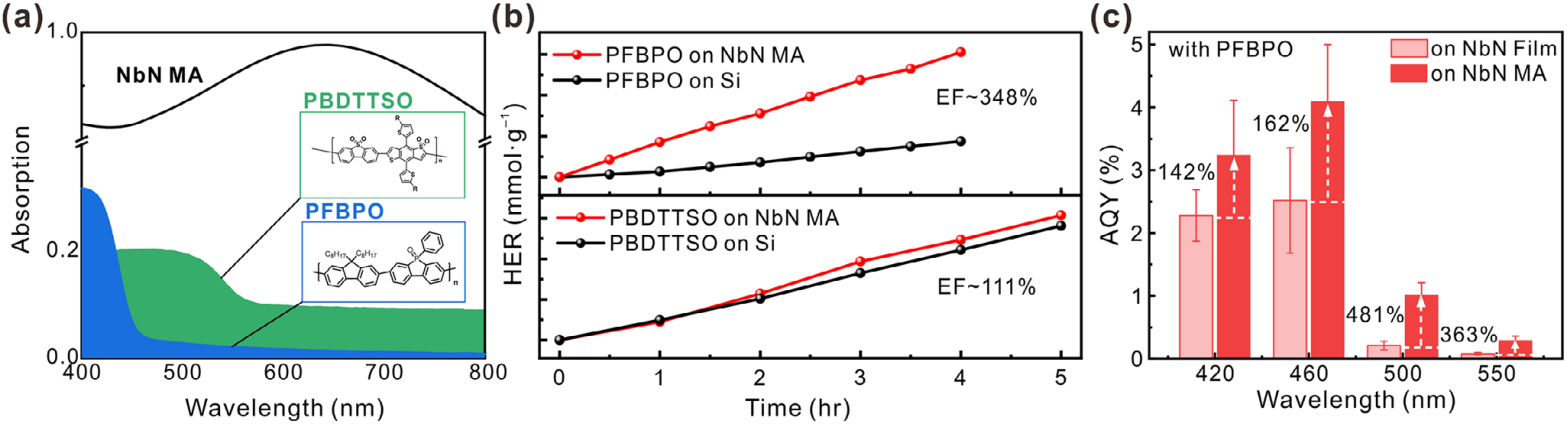
Working principle and apparent quantum yield from the PFBPO/NbN MA. (a) Steady‐state absorption comparison among PFBPO, PBOTTSO, and the NbN MA, highlighting the broadband coverage of the absorber that overlaps with the absorption of the polymer catalysts PFBPO and PBOTTSO. (b) Hydrogen evolution rates of different polymer coatings on the NbN MA and Si substrates, showing the markedly superior activity of PFBPO when integrated with the NbN MA. The enhancement factor (EF) reaches ∼348% for PFBPO and ∼111% for PBDTTSO. (c) Apparent quantum yield (AQY) of PFBPO on the NbN film and NbN MA at different excitation wavelengths within 420–550 nm, revealing a pronounced enhancement on the NbN MA due to broadband light absorption and efficient hot‐carrier injection into the polymer. This results in a maximum AQY improvement of 481% at 500 nm.

To quantify this effect, we measured the AQY of PFBPO on both the planar NbN film and NbN MA substrates (see Supporting Information). As shown in Figure 5c, the AQY of PFBPO on the NbN MA is significantly greater across the entire probed spectral range (420–550 nm), with the most pronounced increase observed near 460 nm (AQY∼ 4.1%), which is just beyond the intrinsic absorption band edge of PFBPO. This peak suggests that plasmonic hot‐carriers generated within the NbN MA are injected into PFBPO and actively participate in the hydrogen evolution reaction, supplementing excitations directly produced by polymer absorption. These results support a model in which the broadband plasmonic modes of NbN efficiently convert incident photons into plasmonic hot carriers through nonradiative plasmon decay. The resulting hot carriers either directly populate the polymer's conduction states or create local electric fields that lower the interfacial barrier, thereby accelerating charge transfer. Even at wavelengths where PFBPO absorbs weakly (>500 nm), the PFBPO/NbN MA exhibits a measurable AQY enhancement of 481% compared with the PFBPO/planar NbN film, demonstrating that the resonant plasmonic metasurface effectively harvests photons and generates hot carriers well beyond the intrinsic absorption band of the polymer. This behavior highlights the role of the plasmonic NbN MA as an active solar energy harvesting platform, where the unique heterostructure band alignment at the NbN/PFBPO interface synergistically broadens the spectral utilization window and enhances the interfacial charge injection efficiency.

## 3 Conclusion

In conclusion, our results reveal that NbN plasmonic MA can efficiently generate and inject hot carriers, leading to a substantial increase in solar‐driven hydrogen evolution. The combination of work‐function‐tunable quasi‐epitaxial NbN films with lithographically defined large‐area MAs enables broadband light absorption exceeding 80% across the visible range and strong near‐field hot spots, which together increase hot‐carrier injection and generation. Ultrafast TAS further confirms sub‐picosecond carrier relaxation (250 fs) and greatly accelerated interfacial charge injection compared with the planar NbN film, directly linking the metasurface architecture to more effective carrier utilization. When integrated with the photocatalyst PFBPO, compared with the unpatterned films, the NbN MA achieves a remarkable 481% increase in hydrogen evolution rate at 500 nm, even beyond the intrinsic absorption range of the polymer. Notably, the PFBPO/NbN MA achieves a remarkable AQY of 4.1% at 460 nm, which represents the highest value reported to date among thin‐film photocatalytic systems (see Table S5) [57, 58, 59]. The calculation results reveal that the enhanced catalytic performance arises primarily from hot‐carrier injection rather than photothermal heating. Finally, the demonstrated approach provides an efficient solar energy harvesting platform for broadband light harvesting and efficient hot‐carrier injection, offering opportunities for scalable solar‐to‐hydrogen conversion, CO_2_ reduction, and other plasmon‐assisted photocatalytic reactions. By establishing transition‐metal nitride metasurfaces as effective hot‐carrier platforms, this work contributes a versatile design strategy that bridges nanophotonics and sustainable energy research and paves the way toward high‐efficiency, durable, and cost‐effective solar‐fuel technologies.

## 4 Methods

### 4.1 Device Fabrication

Transition‐metal nitride (TMN) films were deposited onto single‐crystal sapphire or MgO(100) substrates using a customized high‐vacuum sputtering system. Prior to deposition, the substrates were sequentially cleaned with acetone, isopropanol (IPA), and deionized water under ultrasonic agitation, followed by annealing at 800°C for 1 h under ultrahigh vacuum to remove surface contaminants. Nanodisk arrays with a patterned area of ∼0.6 × 0.6 cm were defined by high‐speed electron‐beam lithography (EBL, ELS‐HS50, ELIONIX INC.). After development, a 50 nm chromium layer was deposited via electron‐beam evaporation to serve as a hard mask. Pattern transfer was achieved by inductively coupled plasma reactive‐ion etching (ICP‐RIE, Oxford) using a Cl_2_/Ar gas mixture, and the Cr hard mask was subsequently removed by wet chemical etching.

### 4.2 Numerical Simulations

Finite‐difference time‐domain (FDTD) simulations were performed using the commercial software Lumerical FDTD Solutions (Lumerical Solutions, Inc.) to calculate the absorption spectra and electric field distributions of the MAs. The experimentally measured refractive index data were first imported and fitted. A plane wave was then used to normally illuminate a single nitride‐based nanodisk on a thin film. Periodic boundary conditions were applied along the in‐plane (x–y) directions to model an infinite array, while perfectly matched layer (PML) boundary conditions were imposed along the z direction. A uniform mesh size of 5 nm was used in all directions to ensure adequate simulation accuracy.

### 4.3 Transient Absorption Spectroscopy Measurements

Time‐resolved transient absorption spectroscopy (TAS) measurements were performed using a FemtoFrame II spectrometer (IB Photonics) controlled by commercial software. The excitation source was a Ti:sapphire femtosecond laser (Tsunami, Spectra‐Physics), with the 80 MHz pulse train amplified to 1 kHz using a chirped‐pulse amplification system (Spitfire, Spectra‐Physics) [53, 54]. The amplified output was split into pump and probe beams via a beamsplitter. The probe beam was focused onto a sapphire crystal to generate a broadband white‐light continuum spanning 450–700 nm, while the pump beam wavelength was tuned using an optical parametric amplifier (TOPAS‐C, Light Conversion). The pump and probe beams were focused onto samples with spot sizes of ∼7 × 10^−4^ cm^2^ and ∼1.6 × 10^−4^ cm^2^, respectively. All optical measurements were conducted at room temperature under controlled relative humidity (40%–50%).

### 4.4 Photocatalytic HER Measurements

The polymer‐coated area was 0.6 × 0.6 cm^2^ fixed by PI tape. Before the photocatalytic H_2_ production experiments, the PI tape was removed, and the polymer film‐coated substrates were immersed in 10 mL of an aqueous solution composed of water, methanol, and triethylamine (TEA) at a 1:1:1 volume ratio. Then, 100 µL of H_2_PtCl_6_ solution (0.5 mg/mL H_2_O) was added to provide the system with the cocatalyst. The solution was degassed by bubbling with Ar for 10 min prior to illumination. The suspension was illuminated with a 350 W Xe lamp (AM 1.5, 1000 W m^−2^) at a constant temperature of 25°C under atmospheric pressure. The H_2_ product gas was collected every 30 min with a gas‐tight syringe and measured using a Shimadzu GC‐2014 gas chromatograph with argon as the carrier gas. Hydrogen was detected by a thermal conductivity detector and quantified based on calibration with standard hydrogen gas concentrations.

## Author Contributions

Y.‐J.L. led the project, conceived the idea, and designed all experimental investigations and data analysis. T.‐Y.P. carried out the experiments, fabrication, numerical simulation, and performed data analysis. T.‐F.H., Y.‐R.Z., and H.‐H.C. contributed to the design and setup of the hydrogen‐evolution and KPFM measurements. T.Y. and T.T. contributed to the large‐area nanofabrication. J.‐W.C. and H.‐Y.K. contributed to the nanofabrication of the plasmonic structures. H.‐W.S. conducted the UPS measurements. T.‐Y.P. drafted the initial version of the manuscript. Y.J.L. revised the manuscript, and all authors discussed the results and commented on the manuscript.

## Conflicts of Interest

The authors declare no conflicts of interest.

## Supporting information




**Supporting File**: advs77436‐sup‐0001‐SuppMat.pdf.

## Data Availability

The datasets analyzed during this study are available from the corresponding author on reasonable request.
