# Supplementary material for: NbN Broadband Plasmonic Absorbers for Efficient Hot‐Carrier Injection Toward Improved Solar‐to‐Hydrogen Conversion
Source: Adv Sci (Weinh). 2026 Aug 24;13(63):e77436. doi: 10.1002/advs.77436 (PMC13502356; doi:10.1002/advs.77436)
Supplement: Supplementary file 1 [file ADVS-13-e77436-s001.pdf]

Supporting information for

NbN Broadband Plasmonic Absorbers for Efficient Hot-Carrier Injection toward Improved Solar-to-Hydrogen Conversion

Tzu-Yu Peng [1,2], Tse-Fu Huang[3], Takeshi Yamaguchi[4], Jia-Wern Chen[1], Hsing-Yu Kuo[1], Ying-Rang Zhuang[3], Hung Wei Shiu[5], Seongmin Im[6], Yang Zhao[6], Takuo Tanaka[4], Ho-Hsiu Chou[3*], and Yu-Jung Lu[1,2*]

[1]*Research Center for Applied Sciences, Academia Sinica, Taipei 11529, Taiwan*

[2]*Graduate Institute of Applied Physics, National Taiwan University, Taipei 10617, Taiwan*

[3]*Department of Chemical Engineering, National Tsing Hua University, Hsinchu 300044, Taiwan*

[4]*Metaphotonics Research Team, RIKEN Center for Advanced Photonics, Saitama 351-0198, Japan*

[5]*National Synchrotron Radiation Research Center (NSRRC), Hsinchu 30076, Taiwan*

[6]*Department of Electrical and Computer Engineering, University of Illinois Urbana-Champaign, Urbana, IL, 61801, USA*

**Corresponding author. Email: yujunglu@gate.sinica.edu.tw ; hhchou@mx.nthu.edu.tw*

Table of contents

Section S1. Fabrication of metasurface absorbers using transition metal nitride

Section S2. Growth parameters for NbN and TiN films

Section S3. Complex permittivity for NbN and TiN films

Section S4. XRD of the optimized NbN and TiN films

Section S5. SEM images of NbN MAs with varying nanodisk diameters

Section S6. Time evolution TAS spectra of NbN and TiN films and MAs

Section S7. Preparation of polymer photocatalyst

Section S8. Color plots of time-resolved TAS for PFBPO deposited on different substrates

Section S9. Kelvin probe force microscopy measurements

Section S10. Fitted carrier lifetime parameters of PFBPO films deposited on different substrates

Section S11. Calculation of the apparent quantum yield (AQY)

Section S12. Spectrum of the 350 W Xe lamp equipped with an AM 1.5 filter

Section S13. Calculation of NbN surface temperature

Section S14. Power-dependent transient absorption spectroscopy measurements

Section S15. Overview of photocatalytic HER performance using different material platforms

Section S1. Fabrication of metasurface absorbers using transition metal nitride

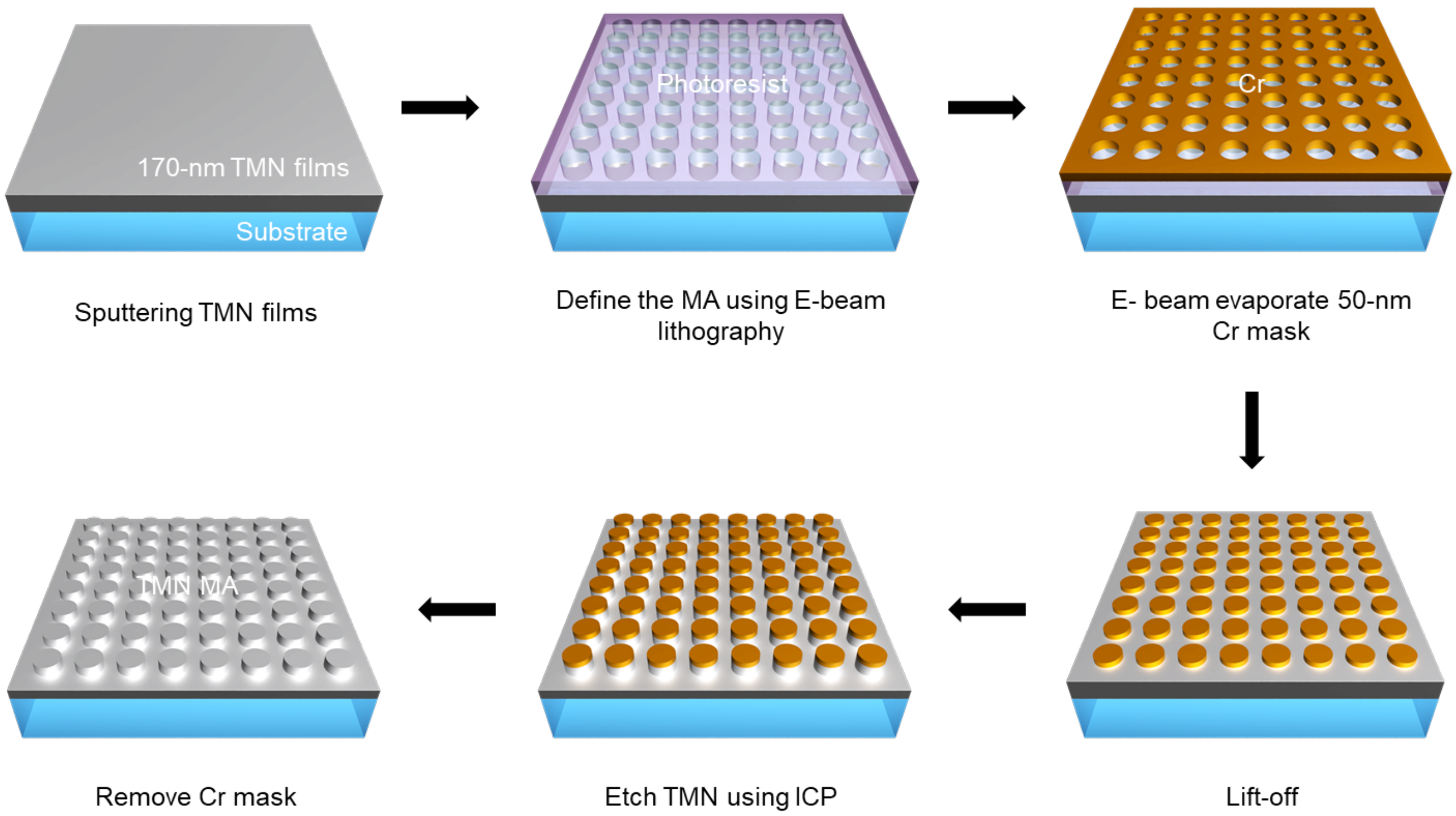

Figure S1. Fabrication process of transition-metal nitride (TMN) metasurface absorbers: Schematic illustration of the step-by-step fabrication flow. A 170-nm-thick TMN film (NbN or TiN) is first deposited on a single-crystal substrate. A photoresist layer is patterned by electron-beam lithography (EBL) to define periodic nanodisk arrays, followed by chromium (Cr) hard-mask deposition. After lift-off, the pattern is transferred into the TMN film via inductively coupled plasma reactive-ion etching (ICP-RIE) to form well-defined nanostructures. The Cr mask is then removed to yield the final TMN metasurface absorber (MA) with highly uniform, lithography-defined nanodisks suitable for broadband plasmonic and photocatalytic studies.

Section S2. Growth parameters for NbN and TiN films

Substrate	**MgO (100)**	**Sapphire (0001)**	**MgO (100)**	**Sapphire (0001)**
Target	NbN (99%)	NbN (99%)	TiN (99%)	TiN (99%)
Base pressure (Torr)	3×10^{-7}	3×10^{-7}	3×10^{-7}	3×10^{-7}
Growth pressure (mTorr)	0.9	0.9	0.9	0.9
Ar (sccm)/N_2 (sccm) flow rate	36:0.1	36:0.1	12:0	12:0
Applied Power (W)	120	120	120	120
Growth Temperature (°C)	800	800	800	800

Table S1. Growth parameters of quasi-epitaxial growth NbN and TiN films films by RF sputtering

Section S3. Complex permittivity for NbN and TiN films

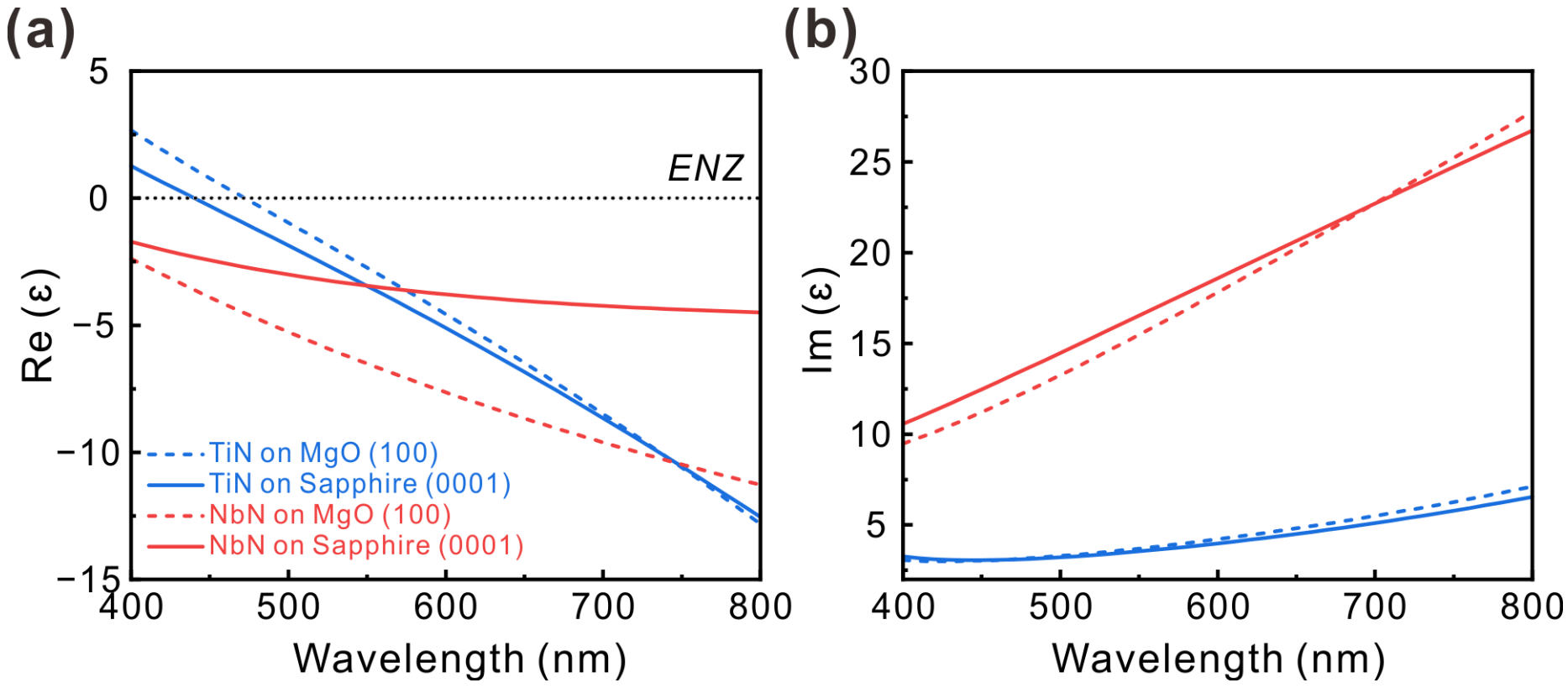

Figure S2. Complex permittivity for NbN and TiN films. a) Real parts and b) imagery part of the dielectric permittivity of TiN and NbN films grown on MgO (100) and sapphire (0001) substrates. Both materials exhibit optically metallic behavior ($\varepsilon_1 < 0$) across the visible–near-IR range, confirming their plasmonic nature. Compared with TiN, NbN shows a more negative ε_1, indicative of stronger free-carrier response. The ENZ wavelength of NbN is blue-shifted relative to TiN, consistent with its higher carrier density and plasma frequency. These characteristics highlight the suitability of NbN for broadband, low-loss plasmonic and hot-carrier applications.

The complex permittivity ε of quasi-epitaxial growth NbN and TiN films can be represented by a combination of the Drude and Lorentz models, based on measurements obtained using variable-angle spectroscopic ellipsometry (VASE, J.A. Woollam Co.)[1].

$$\varepsilon = Re(\varepsilon) + iIm(\varepsilon) = \varepsilon_\infty - \frac{\omega_p^2}{\omega^2 + i\Gamma\omega} + \sum_{j=1}^{n} \frac{f_j \cdot \omega_{0j}^2}{\omega_{0j}^2 - \omega^2 - i\Upsilon_j\omega}$$

In the above equation, complex permittivity ε is a function of the photon frequency. The first term ε_∞ is the high frequency permittivity. The second term corresponds to the Drude model, describing interactions between free electrons and electromagnetic waves, where ω_p denotes the plasma frequency associated with free electrons, and Γ represents the collision rate. The third term consists of Lorentz oscillators, modeling the absorption due to bound electrons, in which f_j is the strength of the oscillator, ω_{0j} is the resonant angular frequency, and Υ_j is the damping factor.

		Drude term (intraband)		Lorentz oscillator 1 (interband)			Lorentz oscillator 2 (interband)		
**TMN**	ε_∞	ω_p (eV)	Γ (eV)	f_1	ω_{o1} (eV)	γ_1	f_2	ω_{o2} (eV)	γ_2
NbN/MgO	3.32	3.31	0.21	557.8	0.51	2.80	2.86	5.12	2.51
NbN/Sapphire	2.20	4.90	0.48	71.4	1.49	4.36	2.94	6.40	3.31
TiN/MgO	3.83	6.84	0.33	1727.3	0.16	10.0	2.16	4.33	0.85
TiN/Sapphire	3.33	6.33	0.34	1739.3	0.16	10.0	2.16	4.21	0.93

Table S2. Fitted parameters based on Drude and Lorentz models for TiN and NbN films grown on MgO (100) and sapphire (0001) substrates.

Section S4. XRD of the optimized NbN and TiN films

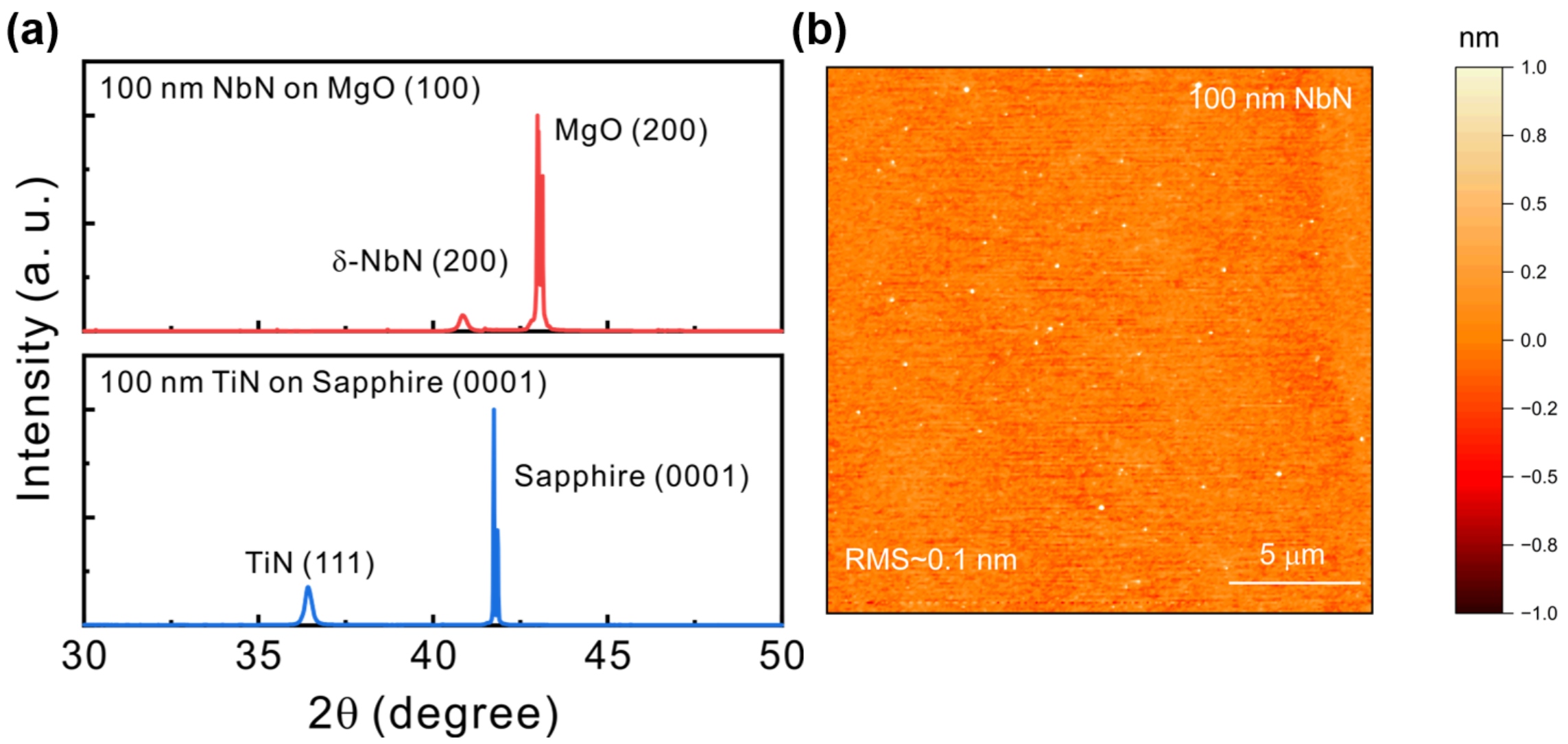

Figure S3. Structural and surface characterization of quasi-epitaxial NbN,TiN thin films**.** a) X-ray diffraction (XRD) patterns of 100-nm-thick NbN film grown on MgO (100) and TiN film grown on sapphire (0001) substrates, showing sharp (200) and (111) reflections indicative of high crystallinity and preferred orientation. The pronounced (200) peak for NbN/MgO confirms quasi-epitaxial growth with minimal lattice mismatch. b) Atomic force microscopy (AFM) image of NbN on MgO (100), revealing an atomically smooth surface with a root-mean-square (RMS) roughness of ~0.1 nm over a 5 µm × 5 µm area. The combination of excellent crystallinity and low surface roughness provides an ideal platform for lithographically defined metasurface fabrication and efficient plasmonic hot-carrier generation.

Section S5. SEM images of NbN MAs with varying nanodisk diameters

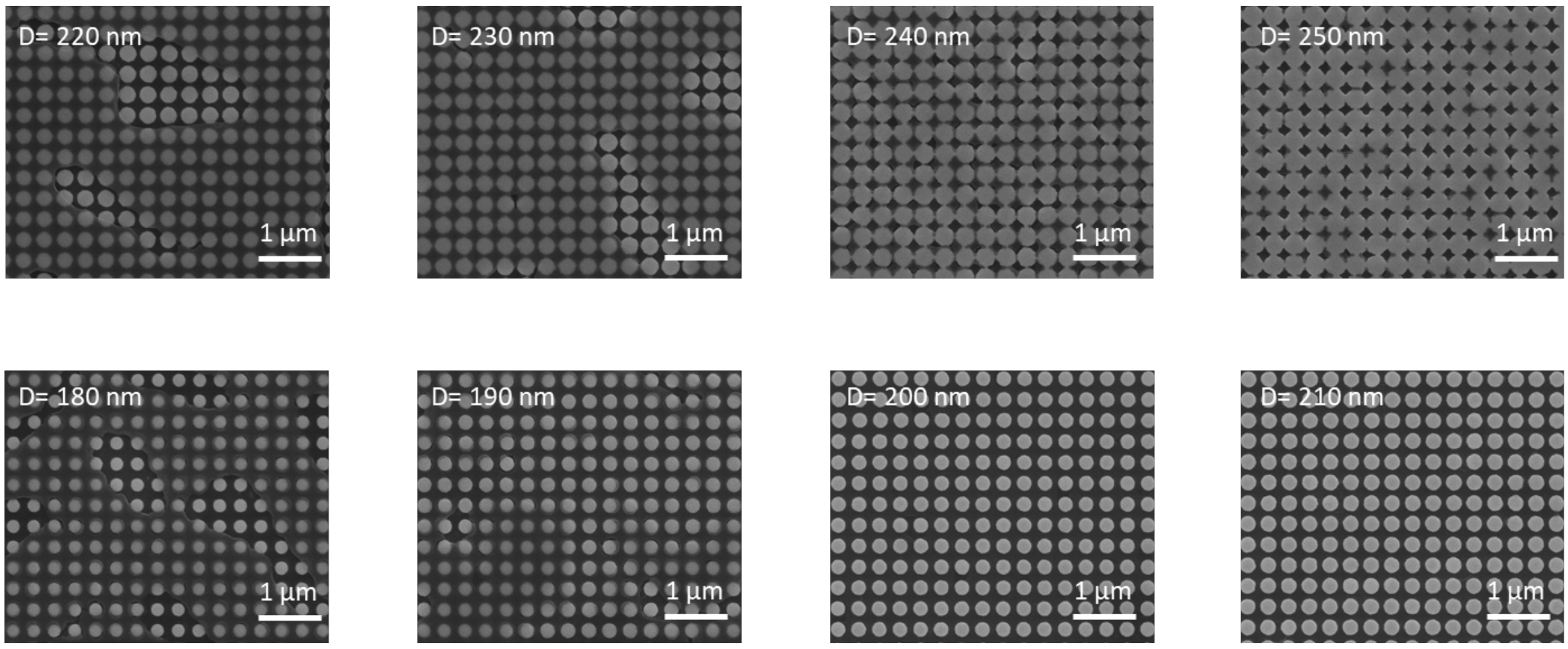

Figure S4. SEM images of NbN metasurface absorbers with different nanodisk diameters. SEM images of NbN metasurface absorbers (MAs) with nanodisk diameters ranging from 180 to 250 nm and with a fixed periodicity of 350 nm.

Section S6. Time evolution TAS spectra of NbN and TiN films and MAs

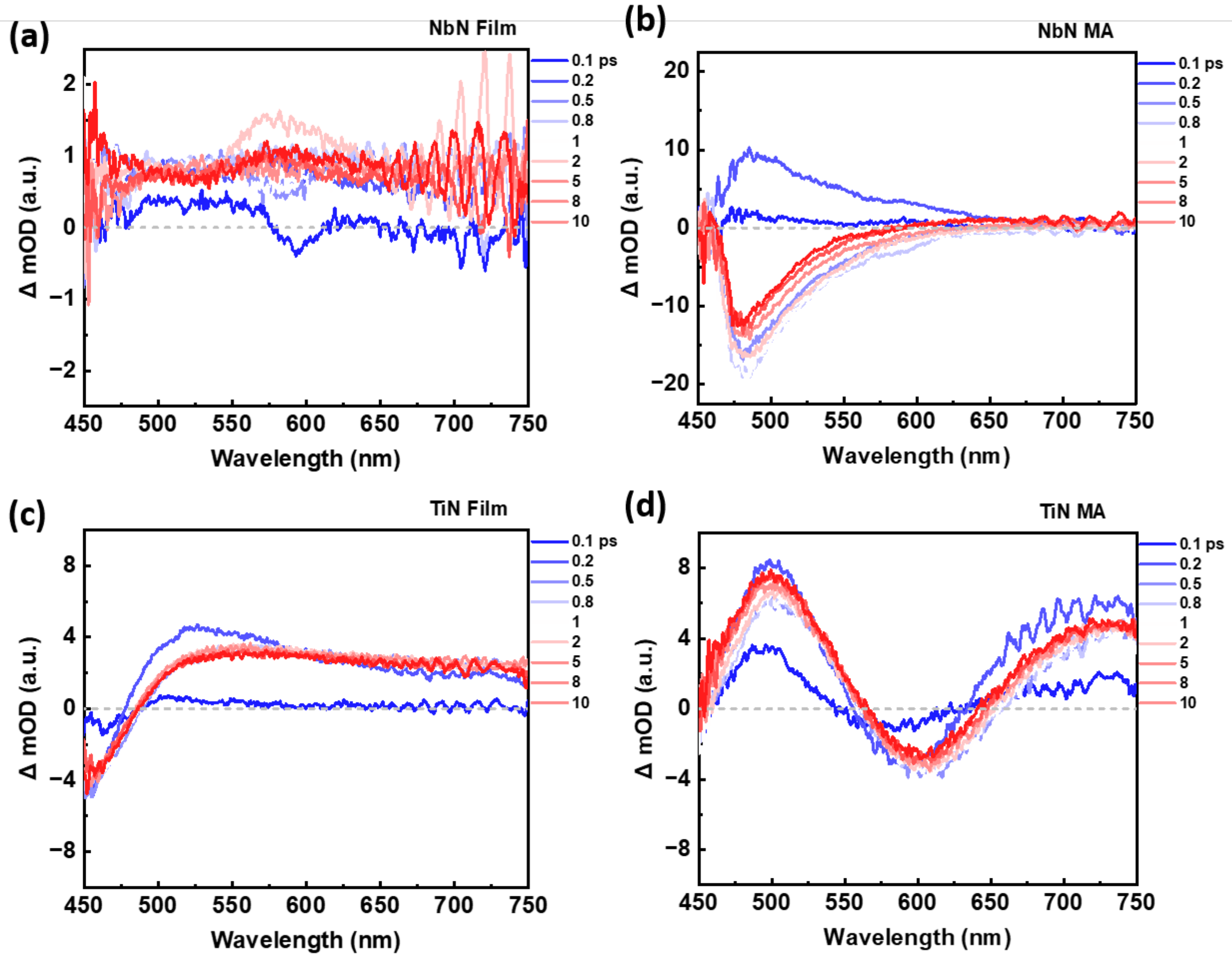

Figure S5. Ultrafast transient absorption dynamics of NbN and TiN films and metasurface absorbers. a) Pump–probe TAS dynamics of NbN film, b) NbN MA c) TiN film d) TiN MA, recorded over probe delays from 0.1 to 10 ps. The NbN MA exhibits the strongest and broadest transient bleaching signal, indicating enhanced plasmon-induced hot-carrier generation and accelerated relaxation dynamics compared with the unpatterned NbN and TiN counterparts.

Section S7. Preparation of polymer photocatalyst

Polymer photocatalyst:

All reagents were obtained from commercial suppliers and used without further purification. Poly[(9,9-dioctyl-9H-fluoren-yl-2,7-diyl)-co-(5-phenylbenzo[b]phosphindole-5-oxide-2,7-diyl)] (PFBPO) was synthesized using previous literature procedures[2].

Monomer F-B (2,7-Bis(4,4,5,5-tetramethyl-1,3,2-dioxaborolan-2-yl)-9,9-di-n-octylfluorene, 643 mg, 1 mmol), bis(3-bromophenyl)(phenyl)phosphine oxide (Br-PPO, 436 mg, 1 mmol), Na_2CO_3 (2540 mg, 24 mmol), tetra-n-butylammonium bromide (TBAB, 13.0 mg, 0.04 mmol), $Pd(PPh_3)_4$ (55.0 mg, 0.048 mmol), toluene (40 mL), and water (10 mL) were used for the polymerization reaction. The mixture was degassed by bubbling with N_2 for 30 minutes and then heated at 120 °C for 72 hours. After cooling to room temperature, bromobenzene was added, and the sealed tube was heated again at 120 °C for 6 hours, followed by the addition of phenylboronic acid and further heating at 120 °C for another 6 hours. The reaction mixture was then cooled to room temperature and poured into MeOH. A yellow-green precipitate formed, which was collected by membrane filtration. The polymer was purified by Soxhlet extraction with MeOH and hexane. Finally, the polymer was dissolved in hot $CHCl_3$, concentrated, and precipitated in MeOH. The resulting polymer was collected and dried under vacuum. ^1H NMR (500 MHz, $CDCl_3$): δ 8.16 (d, J = 7.5 Hz), 7.84 (d, J = 6 Hz), 7.76–7.79 (m), 7.72 (d, J = 7.5 Hz), 7.49–7.58 (m), 7.41–7.45 (m), 7.33–7.36 (m), 1.97 (t, J = 8 Hz), 1.33 (s), 0.99–1.24 (m), 0.72–0.75 (m), 0.60 (br). ^{31}P NMR (202 MHz, $CDCl_3$, 295 K): δ 29.77 (s), 28.11 (s).

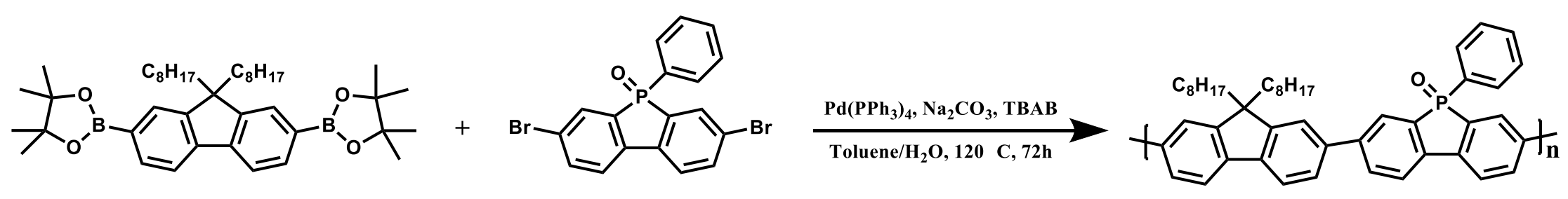

Figure S6. Synthesis procedure of PFBPO.

Section S8. Color plots of time-resolved TAS for PFBPO deposited on different substrates

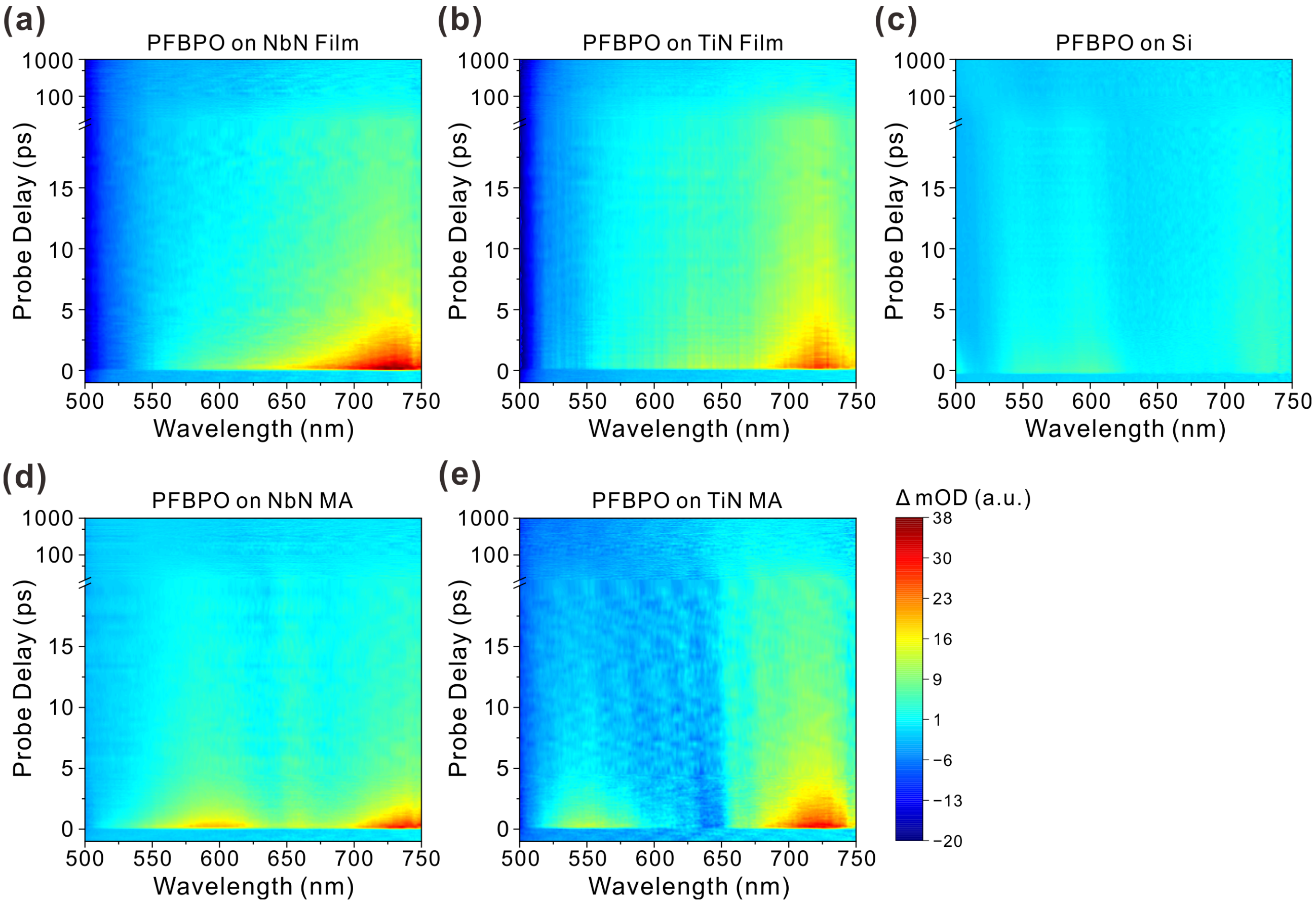

Figure S7. Color plots of time-resolved TAS for PFBPO deposited on different substrates. a) The color plot of the time-resolved TAS for the PFBPO coated on NbN Film b) TiN film c) Si d) NbN MA e) TiN MA.

Section S9. Kelvin probe force microscopy measurements

PFBPO thin films were prepared on NbN, and TiN substrates by spin coating from a PFBPO solution in chlorobenzene with a concentration of 5 mg mL^{-1}. To expose the underlying substrate for direct comparison, a selected region of the PFBPO film was gently removed using an isopropanol-wetted tip, forming a PFBPO/substrate boundary.

Kelvin probe force microscopy (KPFM) measurements were performed using an Asylum Research Cypher S atomic force microscope operated in KPFM mode. Conductive Ti/Ir-coated ASYELEC.02-R2 probes were used, with a nominal spring constant of 42 N m-1 and a resonance frequency of approximately 300 kHz. The scan rate was set between 1 and 2 Hz, and the image resolution was configured at 128 points per line. During the measurements, the tip bias was set to 3 V, and the sample bias was set to 0 V. KPFM images were acquired across the boundaries between the PFBPO-covered and bare substrate regions. The resulting surface-potential maps and corresponding line profiles were used to evaluate the relative surface-potential contrast induced by the deposition of PFBPO on each substrate.

The contact potential difference was defined as:

$$V_{CPD} = V_{Tip} - V_{Sample}$$

where V_{Tip} and V_{Sample} represent the electrostatic potentials of the KPFM tip and sample surface, respectively.

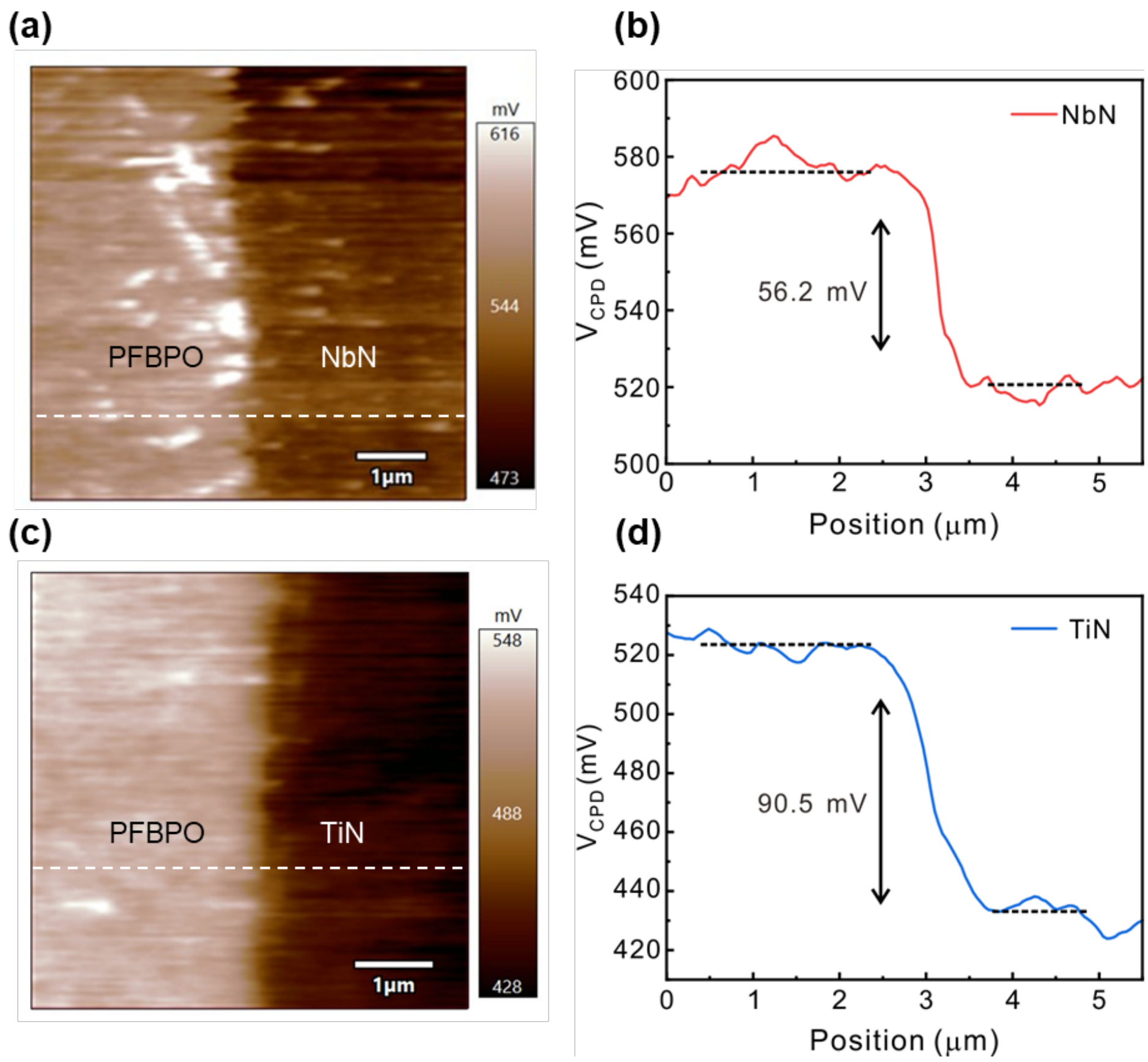

Figure S8. KPFM characterization of the surface-potential contrasts between PFBPO-covered and bare NbN and TiN regions. a) Surface-potential map across the boundary between the PFBPO-covered NbN region and the bare NbN surface. b) Corresponding surface-potential line profile, showing an average potential contrast of approximately 56.2 mV between PFBPO-covered NbN and bare NbN. c) Surface-potential map across the boundary between the PFBPO-covered TiN region and the bare TiN surface. d) Corresponding surface-potential line profile, showing a larger average potential contrast of approximately 90.5 mV between PFBPO-covered TiN and bare TiN.

Section S10. Fitted carrier lifetime of PFBPO films deposited on different substrates

Substrate	A_1	τ_1 (ps)	A_2	τ_2 (ps)
NbN Film	0.69	2.43	0.31	28.9
NbN MA	0.76	1.92	0.22	23.0
TiN Film	0.50	3.13	0.43	46.5
TiN MA	0.62	2.01	0.34	30.5
Si	*NA*	*NA*	*NA*	69.3

Table S3. Table of fitted carrier lifetime of PFBPO films deposited on different substrates.

The time-resolved TAS spectra is fitted using the two exponential decay model as following equation:

$$y = y_0 + \frac{A_1}{t_1} e^{-\frac{x}{t_1}} + \frac{A_2}{t_2} e^{-\frac{x}{t_2}}$$

Where t_i and A_i represent each decay time and its weight, respectively, and y_0 represent the base line.

Section S11. Calculation of the apparent quantum yield (AQY)

The entire hydrogen evolution reaction is assumed to be a two-electron process. The AQY values were calculated using the following equation:

$$AQY(\%) = \frac{2 \times the\ number\ of\ produced\ hydrogen\ molecules}{the\ number\ of\ incident\ photons}$$

$$= \frac{2 \times \hbar \times H \times N_A}{P \times t \times A \times \frac{\lambda}{c}} \times 100\%$$

Here, H is the amount of hydrogen produced (in units of mol), $\hbar$ *is* the Planck constant, N_A is the Avogadro constant, A is the irradiation area (m^2), P is the intensity of the monochromatic light (W m^{-2}), t is the time (s), λ is the wavelength of the monochromatic light (m), and c is the speed of light ($m \cdot s^{-1}$).

Section S12. Spectrum of the 350 W Xe lamp equipped with an AM 1.5 filter

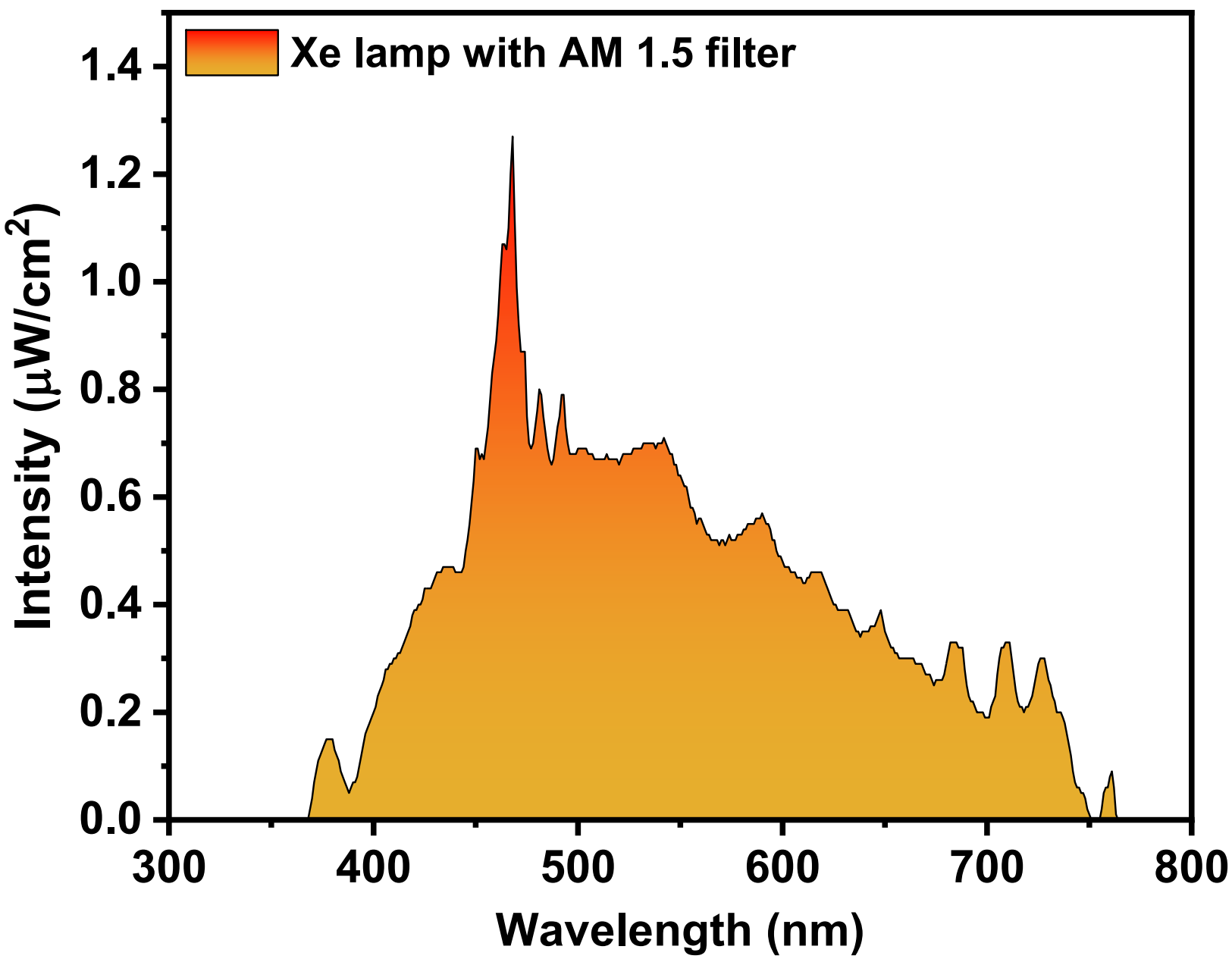

Figure S9. Spectrum of the 350 W xenon (Xe) lamp equipped with an AM 1.5 filter, used as the simulated solar illumination source for photocatalytic HER measurements.

Section S13. Calculation of NbN surface temperature

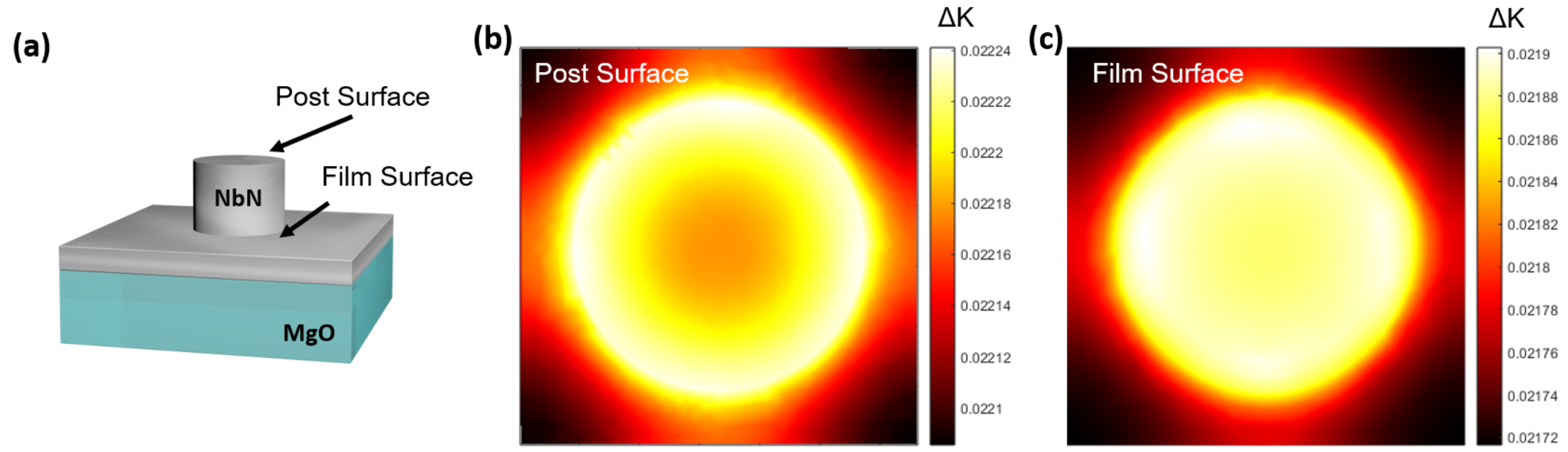

Figure S10. Calculated surface temperature distribution of the NbN metasurface absorber under AM 1.5 illumination. a) Schematic of the NbN metasurface absorber consisting of NbN nanodisks fabricated on a NbN film. The post surface and film surface are indicated. b) Simulated surface temperature distribution on the NbN post surface. c) and beneath the nanodisk structure, showing negligible temperature variation.

Section S14. Power-dependent transient absorption spectroscopy measurements

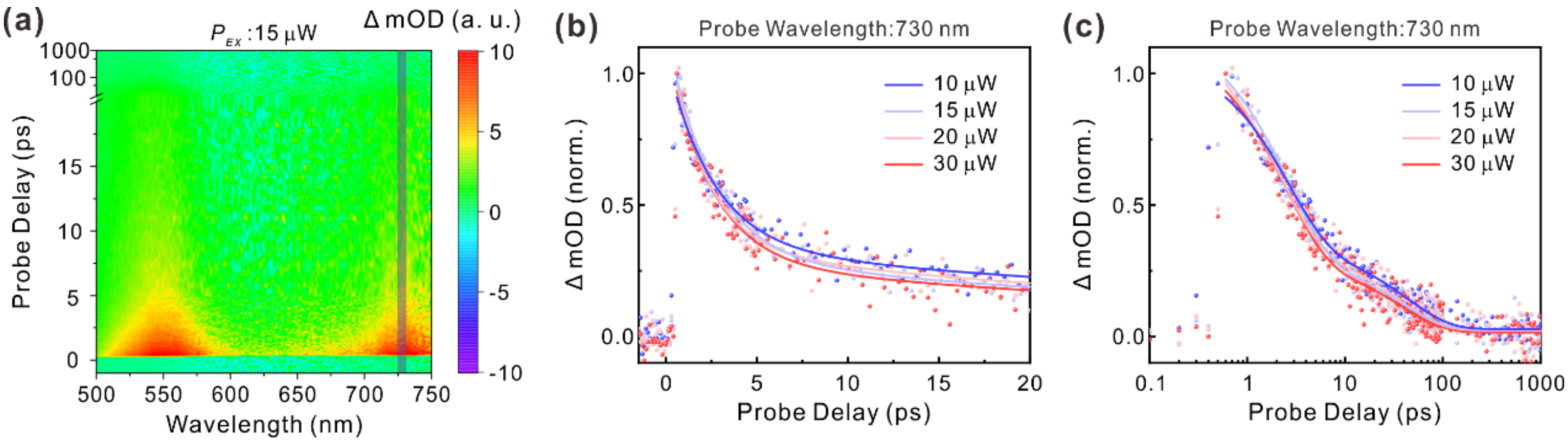

Figure S11. Power-dependent transient absorption spectroscopy measurements of PFBPO on the NbN metasurface absorber. a) Transient absorption map measured at an excitation power of 15 μW. b) Normalized transient absorption decay profiles monitored at a probe wavelength of 730 nm under excitation powers ranging from 10 to 30 μW. The solid curves represent the corresponding fitting results. c) The same decay traces plotted on a logarithmic time scale. The nearly overlapping decay dynamics over the measured excitation-power range indicate that the carrier-relaxation process remains essentially unchanged with increasing excitation power, suggesting that photothermal heating is not the dominant mechanism.

Excitation Power	A_1	τ_1 (ps)	A_2	τ_2 (ps)
10 μW	0.72	2.56 ± 0.20	0.31	43.9 ± 4.5
15 μW	0.86	2.52 ± 0.18	0.27	37.8 ± 4.8
20 μW	0.84	2.08 ± 0.16	0.31	36.0 ± 3.9
30 μW	0.84	2.40 ± 0.17	0.27	38.1 ± 4.8

Table S4. Fitted carrier lifetimes of PFBPO on the NbN metasurface absorber measured at different excitation powers.

Section S15. Overview of photocatalytic HER performance using different material platforms

Photocatalyst	Source	Intensity (mW cm^{-2})	Amount	HER (μmol h^{-1} g^{-1})	AQY Max	AQY@ 500 nm
Au/TiO_2[3]	Xe	500	50 mg	0.5	0.0003	N.A.
Ag@C_3N_4 core–shell[4]	Xe	500 W	100 mg	25.2	N.A.	N.A.
Pt@MOF/Au[5]	Xe	300 W	5 mg	1743	N.A.	N.A.
AuNR/TiO_2[6]	visible light	N.A.	1 mg	11.6	N.A.	N.A.
3SCN/Fluorescein [7]	visible light	N.A.	5 mg	2014.2	N.A.	N.A.
Au/CdSe-0.5 NCs[8]	Xe	2000	1 mg	48800	0.95@420 nm	0.5
Ag/CQDs/g-C_3N_4[9]	visible light	N.A.	5 mg	626.93	4.81@400 nm	N.A.
MoO_6/CdS-DETA[10]	Xe	N.A.	50 mg	7370	20.7@420 nm	N.A.
Ag/$ZnIn_2S_4$/TiO_2 [11]	AM1.5	1000	5 mg	6.6	0.2@420 nm	0.1
UCNPs-Pt@MOF/Au[12]	AM1.5	N.A.	10 mg	280	N.A.	N.A.
Ag/g-C_3N_4/TiO_2 NFs[13]	AM1.5	1000	5 mg	300	N.A.	N.A.
2DPA[14]	AM1.5	1000	100 mg	10084.6	6.5@400 nm	4.2
AuP25-DP[15]	320-500	N.A.	50 mg	33000	N.A.	N.A.
Au/P25 TiO_2[16]	UV	65	6.5 mg	34200	N.A.	N.A.
CQDs Cu/CQDs[17]	Solar light	N.A.	0.15 mg	64000	N.A.	N.A.
Au-chain@ $Zn_{0.67}Cd_{0.33}S$[18]	>420	N.A.	100 mg	16420	54.6@420 nm	33
Pt@Au NR769/ CNNT650[19]	>420	N.A.	20 mg	10350	9.1@420 nm	N.A.
Au/Cu_2ZnSnS_4 core/shell NPs[20]	AM1.5	1000	10 mg	102	N.A.	N.A.
HM-TiPPh[21]	Solar light	N.A.	100 mg	1873	N.A.	N.A.
Au/TiO_2-anatase[22]	>360	1800	50 mg	13300	N.A.	N.A.
This work	AM1.5	1000	0.2 mg	7340	4.1 @450 nm	1.01

Table S5. Overview of photocatalytic HER performance using different material platforms, comparing the activity of various plasmonic and non-plasmonic systems.

References

[1] J. N. Hilfiker, N. Singh, T. Tiwald, *et al.*, "Survey of Methods to Characterize Thin Absorbing Films with Spectroscopic Ellipsometry", *Thin Solid Films* **516**, no. 22 (2008): 7979–7989. https://doi.org/10.1016/j.tsf.2008.04.060

[2] C.-L. Chang, W.-C. Lin, L.-Y. Ting, *et al.,* "Main-Chain Engineering of Polymer Photocatalysts with Hydrophilic Non-Conjugated Segments for Visible-Light-Driven Hydrogen Evolution", *Nature Communications* **13**, no. 1 (2022): 5460. https://doi.org/10.1038/s41467-022-33211-1

[3] Z. Bian, T. Tachikawa, P. Zhang, M. Fujitsuka, T. Majima, "Au/TiO_2 Superstructure-Based Plasmonic Photocatalysts Exhibiting Efficient Charge Separation and Unprecedented Activity", *Journal of the American Chemical Society* **136**, no. 1 (2014): 458–465. https://doi.org/10.1021/ja410994f

[4] X. Bai, R. Zong, C. Li, *et al.*, "Enhancement of Visible Photocatalytic Activity *via* Ag@C_3N_4 Core–Shell Plasmonic Composite", *Applied Catalysis B: Environmental* **147**, (2014): 82–91. https://doi.org/10.1016/j.apcatb.2013.08.007

[5] J. D. Xiao, L. Han, J. Luo, S. H. Yu, H. L. Jiang, "Integration of Plasmonic Effects and Schottky Junctions into Metal–Organic Framework Composites: Steering Charge Flow for Enhanced Visible-Light Photocatalysis", *Angewandte Chemie International Edition* **57**, no. 4 (2018): 1103–1107. https://doi.org/10.1002/anie.201711725

[6] B. Wu, D. Liu, S. Mubeen, *et al.,* "Anisotropic Growth of TiO_2 onto Gold Nanorods for Plasmon-Enhanced Hydrogen Production from Water Reduction", *Journal of the American Chemical Society* **138**, no. 4 (2016): 1114–1117. https://doi.org/10.1021/jacs.5b11341

[7] J. Qin, J. Huo, P. Zhang, *et al.*, "Improving the Photocatalytic Hydrogen Production of Ag/gC_3N_4 Nanocomposites by Dye-Sensitization under Visible Light Irradiation", *Nanoscale* **8**, no. 4 (2016): 2249–2259. https://doi.org/10.1039/C5NR06346A

[8] R. Shi, Y. Cao, Y. Bao, *et al.*, "Self-Assembled Au/CdSe Nanocrystal Clusters for Plasmon-Mediated Photocatalytic Hydrogen Evolution", *Advanced Materials* **29**, no. 27 (2017): 1700803. https://doi.org/10.1002/adma.201700803

[9] J. Qin, H. Zeng, "Photocatalysts Fabricated by Depositing Plasmonic Ag Nanoparticles on Carbon Quantum dots/Graphitic Carbon Nitride for Broad Spectrum Photocatalytic Hydrogen Generation", *Applied Catalysis B: Environmental* **209**, (2017): 161–173. https://doi.org/10.1016/j.apcatb.2017.03.005

[10] J. Lv, J. Zhang, J. Liu, *et al.*, "Bi SPR-promoted Z-scheme Bi_2MoO_6/CdS-Diethylenetriamine Composite with Effectively Enhanced Visible Light Photocatalytic Hydrogen Evolution Activity and Stability", *ACS Sustainable Chemistry & Engineering* **6**, no. 1 (2018): 696–706. https://doi.org/10.1021/acssuschemeng.7b03032

[11] Z. Zhang, Y. Huang, K. Liu, *et al.*, "Multichannel-Improved Charge-Carrier Dynamics in Well-Designed Hetero-Nanostructural Plasmonic Photocatalysts Toward Highly Efficient Solar-to-Fuels Conversion", *Advanced Materials* **27**, no. 39 (2015): 5906–5914. https://doi.org/10.1002/adma.201502203

[12] D. Li, S. H. Yu, H. L. Jiang, "From UV to Near-Infrared Light-Responsive Metal–Organic Framework Composites: Plasmon and Upconversion Enhanced Photocatalysis", *Advanced Materials* **30**, no. 27 (2018): 1707377. https://doi.org/10.1002/adma.201707377

[13] X. Wei, C. Shao, X. Li, *et al.*, "Facile *in situ* Synthesis of Plasmonic Nanoparticles-Decorated gC_3N_4/TiO_2 Heterojunction Nanofibers and Comparison Study of Their Photosynergistic Effects for Efficient Photocatalytic H_2 Evolution", *Nanoscale* **8**, no. 21 (2016): 11034–11043. https://doi.org/10.1039/C6NR01491G

[14] Z. Lin, C. Du, B. Yan, C. Wang, G. Yang, "Two-Dimensional Amorphous NiO As A Plasmonic Photocatalyst for Solar H_2 Evolution", *Nature Communications* **9**, no. 1 (2018): 4036. https://doi.org/10.1038/s41467-018-06456-y

[15] J. B. Priebe, J. r. Radnik, A. J. Lennox, *et al.*, "Solar Hydrogen Production by Plasmonic Au–TiO_2 Catalysts: Impact of Synthesis Protocol and TiO_2 Phase on Charge Transfer Efficiency and H_2 Evolution Rates", *ACS Catalysis* **5**, no. 4 (2015): 2137–2148. https://doi.org/10.1021/cs5018375

[16] V. Jovic, W.-T. Chen, D. Sun-Waterhouse, *et al.*, "Effect of Gold Loading and TiO_2 Support Composition on the Activity of Au/TiO_2 Photocatalysts for H_2 Production from Ethanol–Water Mixtures", *Journal of Catalysis* **305**, (2013): 307–317. https://doi.org/10.1016/j.jcat.2013.05.031

[17] P. Zhang, T. Song, T. Wang, H. Zeng, "*In-situ* Synthesis of Cu Nanoparticles Hybridized with Carbon Quantum Dots as a Broad Spectrum Photocatalyst for Improvement of Photocatalytic H_2 Evolution", *Applied Catalysis B: Environmental* **206**, (2017): 328–335. https://doi.org/10.1016/j.apcatb.2017.01.051

[18] G. Yu, J. Qian, P. Zhang, *et al.*, "Collective Excitation of Plasmon-Coupled Au-Nanochain Boosts Photocatalytic Hydrogen Evolution of Semiconductor", *Nature Communications* **10**, no. 1 (2019): 4912. https://doi.org/10.1038/s41467-019-12853-8

[19] L. Zhang, N. Ding, L. Lou, *et al.*, "Localized Surface Plasmon Resonance Enhanced Photocatalytic Hydrogen Evolution *via* Pt@Au NRs/C_3N_4 Nanotubes under Visible-Light Irradiation", *Advanced Functional Materials* **29**, no. 3 (2019): 1806774. https://doi.org/10.1002/adfm.201806774

[20] E. Ha, L. Lee, J. Wang, *et al.*, "Significant Enhancement in Photocatalytic Reduction of Water to Hydrogen by Au/Cu_2ZnSnS_4 Nanostructure", *Advanced Materials (Deerfield Beach, Fla.)* **26**, no. 21 (2014): 3496–3500. https://doi.org/10.1002/adma.201400243

[21] H. Li, Y. Sun, Z. Y. Yuan, Y. P. Zhu, T. Y. Ma, "Titanium Phosphonate Based Metal–Organic Frameworks with Hierarchical Porosity for Enhanced Photocatalytic Hydrogen Evolution", *Angewandte Chemie International Edition* **57**, no. 12 (2018): 3222–3227. https://doi.org/10.1002/anie.201712925

[22] W.-T. Chen, A. Chan, Z. H. Al-Azri, *et al.*, "Effect of TiO_2 Polymorph and Alcohol Sacrificial Agent on the Activity of Au/TiO_2 Photocatalysts for H_2 Production in Alcohol–Water Mixtures", *Journal of Catalysis* **329**, (2015): 499–513. https://doi.org/10.1016/j.jcat.2015.06.014
